# Surfactant-Driven Effects on the Antifungal Activity of *Lippia origanoides* Kunth Essential Oil Encapsulated in Lipid-Based Nanosystems

**DOI:** 10.1021/acsomega.4c08578

**Published:** 2025-02-20

**Authors:** Gabriela Alberto Gil, Letícia Kakuda, Ludmilla Tonani, Marcia Regina von Zeska Kress, Wanderley Pereira Oliveira

**Affiliations:** School of Pharmaceutical Sciences of Ribeirão Preto, University of São Paulo, Ribeirão Preto 14040-903, Brazil

## Abstract

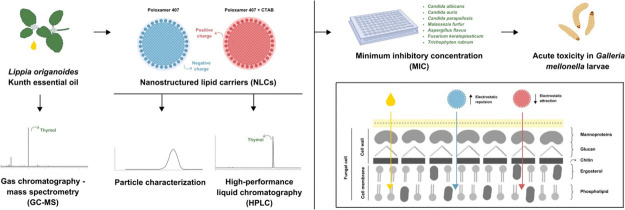

In recent decades, the recurrence and mortality rates of fungal infections have increased, likely due to antifungal resistance and insufficient attention from healthcare authorities. This trend highlights the urgent need for new antifungal treatments, with essential oils (EOs) emerging as promising alternatives. This study focuses on the characterization, nanoencapsulation, and evaluation of the EO of *Lippia origanoides* Kunth - pepper-rosemary - on toxicity and antifungal activity against filamentous fungi and yeasts, with a particular emphasis on the influence of surfactants. The EO was characterized by GC–MS and encapsulated in Nanostructured Lipid Carriers (NLCs) using either a nonionic surfactant or a combination with a cationic surfactant. NLCs were further characterized by the determination of the retention of the marker compound by high-performance liquid chromatography (HPLC), of morphology by transmission electronic microscopy (TEM), and their stability was assessed under thermal stress over 28 days. Minimum inhibitory concentrations (MIC) were determined against four yeast fungi - *Candida albicans* (ATCC 64548), *Candida auris* (CDC B11903), *Candida parapsilosis* (ATCC 22019), and *Malassezia furfur* (ATCC 14521) - and three filamentous fungi - *Aspergillus flavus* (ATCC 204304), *Fusarium keratoplasticum* (ATCC 36031), and *Trichophyton rubrum* (ATCC 28188). The NLC’s acute toxicity was evaluated in *Galleria mellonella* larvae. The results demonstrated the stability, safety, and potent antifungal efficacy of EO-loaded NLCs. The charge of the NLCs played a critical role in their antifungal performance for most fungal species. The differential responses observed suggest that CTAB enhances antifungal activity by imparting a positive charge to the nanoparticles, creating an additive effect with thymol. CTAB’s ability to reverse the fungal cell surface charge from negative to positive was significant. However, *C. auris*, *A. flavus*, and *F. keratoplasticum* showed no sensitivity to CTAB, indicating that surface charge was not a factor for these fungi. The EO and its NLC formulations exhibited significant in vitro antifungal activity, suggesting their potential as alternative therapies for fungal infections.

## 1 Introduction

In the last few decades, fungal infections have become increasingly recurrent, affecting over 1 billion people annually, with 150 million severe cases and 1.7 million fatalities.^1,2^ These numbers are expected to increase substantially owing to the growing population of vulnerable groups, such as the elderly, critically ill, and immunocompromised patients.^1,3^

Despite their potential risks, healthcare authorities have consistently underestimated and neglected fungal pathologies, resulting in increased cases of cross-resistance to existing antifungal treatments.^1−5^ Several antifungals are available for treating superficial and systemic infections, primarily categorized into azoles, echinocandins, and polyenes.^6^ However, the emergence of species with inherent multiresistance (e.g., *Candida auris*) or strains with acquired resistance (e.g., *Aspergillus* spp.) has increased in recent years. These developments have resulted in treatment challenges, the evolution of superficial to systemic infections, and a subsequent rise in mortality rates.^1,6,7^

In this context, the need to discover effective antifungals against resistant species.^1,3^ Given the longstanding use of plants in medicinal practices throughout human history, essential oils (EOs) have emerged as promising candidates due to their notable antifungal activity. With their rich composition of active ingredients, EOs can act by various mechanisms of action and potentially mitigate the development of resistance phenomena.^8^

*Lippia origanoides* Kunth (syn. *Lippia sidoides* Cham., from the botanical family Verbenaceae J.St.-Hil.), commonly known as pepper-rosemary, is a plant native to the semiarid regions of Brazil. The EO extract from the leaves of this plant exhibits antifungal and anti-inflammatory properties, primarily attributed to the presence of the monoterpene thymol and its isomer, carvacrol.^9,10^ Thymol is a hydrophobic compound that interacts with fungal cell membranes, inducing cell death and disrupting biofilms, thereby minimizing fungal resistance and reducing inflammatory signals by suppressing cyclooxygenase-2 (COX-2) and inhibiting cytokines and inflammatory mediators’ production.^11^

Despite their biological potential, using EOs in their native form can lead to the volatilization or oxidation of compounds, skin sensitization, and systemic intoxication.^12,13^ Moreover, its inclusion in pharmaceutical formulations can be challenging due to its liquid form and hydrophobic nature. Nanotechnology, particularly nanostructured lipid carriers (NLCs), composed of a blend of solid and liquid lipids stabilized with surfactants, can contribute to improved stability and bioactivity of the active ingredients, as well as promote sustained release and facilitate incorporation into stable, safe, and effective topical formulations.^14,15^ The appropriate selection of components, particularly surfactants, can enhance the biological activity of the encapsulated compounds.^16^

Hence, the objective of the present study was to characterize and encapsulate the pepper-rosemary EO in NLCs, assessing the influence of the nanocarriers’ surface charge on their physicochemical stability, acute toxicity in a *Galleria mellonella* larvae model, and antifungal activity against four yeast fungi - *Candida albicans* (ATCC 64548), *Candida auris* (CDC B11903), *Candida parapsilosis* (ATCC 22019), and *Malassezia furfur* (ATCC 14521) - and three filamentous fungi - *Aspergillus flavus* (ATCC 204304), *Fusarium keratoplasticum* (ATCC 36031), and *Trichophyton rubrum* (ATCC 28188). A comprehensive review of some aspects of the fungal species investigated and their potential to cause human diseases is presented as Table 1.

**Table 1:** Fungal Species Addressed in the Study and Their Potential to Cause Human Diseases

*Candida* spp.	*Candida* spp. are opportunistic pathogens that colonize the skin and mucous membranes. These yeasts have the potential to induce superficial infections, as well as invasive systemic infections and candidemia, particularly in immunocompromised patients.^17^ It is estimated that 90% of invasive infections are caused by *Candida* spp., with a mortality rate ranging from 20 to 40%.^4,18^*Candida albicans* is the most common species found in both adult and pediatric patients, representing nearly 50% of infection cases.^18^ Its prevalence is attributed to its capacity to switch morphology, produce biofilms, and exhibit resistance mechanisms against azole and echinocandin antifungals.^5^ However, in recent decades, the misuse of antimicrobials and the increase in vulnerable populations have contributed to a considerable increase in cases associated with *Candida parapsilosis* and other non-*albicans Candida* spp.^18^ Since 2009, *Candida auris* has attracted the attention of the medical and scientific community due to its multidrug resistance, rapid global emergence, and high mortality rates.^17^
*Malassezia furfur*	*Malassezia* spp. is lipid-dependent fungi that comprise the normal microbiome of human skin, particularly in areas rich in sebaceous glands such as the face, trunk, and scalp.^19,20^*Malassezia* spp. account for approximately 80% of the fungal population on human skin.^21^ Although they are essential for skin protection, increased sebum production, dysregulation of the cutaneous microbiome, and the presence of skin lesions can favor the occurrence of *Malassezia* spp. infections.^22^ Their presence in imbalance has been associated with the occurrence of superficial infections, including seborrheic dermatitis, dandruff, psoriasis, acne, and folliculitis, as well as invasive infections in catheter-using patients and immunocompromised individuals, particularly neonates.^20,23^
*Aspergillus flavus*	*Aspergillus flavus* is a filamentous fungus typically found in soil and is an important agricultural pathogen, notably impacting maize, peanut, and cottonseed crops. Beyond inflicting substantial economic losses, the contamination of foods with *A. flavus* spores and their production of aflatoxins serves as a prominent conduit for intoxication (aflatoxicosis) and infection (aspergillosis).^24^ Aflatoxicosis is an intoxication caused by the ingestion of foods contaminated with aflatoxins, especially aflatoxin B1, a secondary metabolite of *A. flavus* with carcinogenic and mutagenic properties.^24,25^ As a result, acute and chronic intoxication can lead to hepatotoxicity and liver cancer, respectively.^25^ Conversely, aspergillosis is an infectious process resulting from inhalation of *Aspergillus* spp. spores, which typically remain localized to the lungs but can spread to extrapulmonary sites in immunocompromised individuals.^26,27^
*Fusarium keratoplasticum*	*Fusarium* spp. are filamentous fungi found throughout the environment (e.g., air, water, and soil).^28^ In particular, *Fusarium keratoplasticum* is commonly found in water pipes and stands out as the most important opportunistic pathogen within the *Fusarium solani* Species Complex (FSSC). It is notably linked to both superficial and invasive infections, which are particularly prevalent in immunocompromised patients (.^29^ Furthermore, *Fusarium* spp. infections typically exhibit resistance to conventional antifungal treatments, which poses challenges in the management of these infections and leads to increased mortality rates.^30^
*Trichophyton rubrum*	Dermatophytes are pathogenic and keratolytic fungi that infect the skin, nails, and scalp of humans and animals, causing superficial infections.^31,32^ While dermatomycoses are generally not fatal, they are associated with discomfort and reduced quality of life.^33^ The most important dermatophyte is *Trichophyton rubrum*, a strict human pathogen responsible for causing *Tinea pedis*.^23,33^ Though primarily targeting superficial areas, *T. rubrum* can invade the dermis, subcutaneous tissue, and hair follicles.^33^ While *T. rubrum* infections are generally manageable, the frequent recurrence and the use of antimicrobials at subinhibitory concentrations contribute to the emergence of resistance.^34^

## 2 Materials and Methods

### 2.1 Characterization of *Lippia origanoides* Essential Oil

The EO was purchased from PRONAT (Produtos Naturais LTDA, Brazil) and registered in SisGen (A452930). The major components of the EO were identified using gas chromatography coupled with mass spectrometry (GC–MS; Shimadzu, model GCMS QP-2010, Japan). The analyses were performed on a capillary column EN5–MS (30 m × 0.25 mm × 0.25 μm) using hydrogen as the carrier gas at a 1.0 mL/min flow rate. The temperature program started at 60 °C and increased to 240 °C at 3 °C/min. The EO components were identified by comparing the mass spectrometry data with entries in the Wiley Online Library, and the linear retention index (IRL) was calculated using a homologous series of alkanes (C8–C20) as reported by Adams (2007).^35^

### 2.2 Formulation and Stability Evaluation of the Nanostructured Lipid Carriers

The hot homogenization method was used to develop NLCs.^36,37^ The raw materials of the formulations were listed according to the standard and the International Union of Pure and Applied Chemistry (IUPAC) nomenclature. Thymol (5-methyl-2-(propan-2-yl)phenol) and Cetrimonium Bromide (CTAB; hexadecyl(trimethyl)azanium;bromide) were purchased from Sigma-Aldrich (Germany). The Glyceryl Distearate ((2-hydroxy-3-octadecanoyloxypropyl) octadecanoate) with a melting point of 50–60 °C was kindly donated by Gattefossé (France), Oleic Acid ((Z)-octadec-9-enoic acid) from Synth (Brazil), Poloxamer 407 (2-methyloxirane;oxirane) from BASF (USA), and Aminomethyl Propanol (AMP 95; 2-amino-2-methylpropan-1-ol) from Angus Chemical (USA).

The lipid phase was heated to approximately 10 °C above the melting point of the solid lipid. The EO was added only after the solid’s lipid melting and had been previously acclimatized to prevent the solidification of Glyceryl Distearate while Thymol was heated with the lipid phase. The surfactant Poloxamer 407, or CTAB, was solubilized in the aqueous phase. The aqueous phase was heated to 70 °C and then mixed into the lipid phase. Subsequently, the system was homogenized using an UltraTurrax (IKA model T18 basic, USA) at 22,000 rpm for 3 min, followed by ultrasonic processing (SONICS Vibracell model CV334, USA) at 45% amplitude for six cycles of 5 min on and 2 min off, while keeping the formulation in iced water. After 24 h, AMP 95 (Angus Chemical, USA) was added to the F2 group formulation to adjust the pH, making it compatible with skin pH (4.1–5.8).^38^ The composition of each developed NLC formulation is presented in Table 2.

**Table 2:** Compositions of the Nanostructured Lipid Carriers (NLCs) Investigated (% w/w)

constituents (IUPAC nomenclature)^a^	F1_B_	F1_T_	F1_EO_	F2_B_	F2_T_	F2_EO_
glyceryl distearate^b^	5%	5%	5%	5%	5%	5%
oleic acid^b^	1%	1%	1%	1%	1%	1%
poloxamer 407^c^	4%	4%	4%	3%	3%	3%
cetrimonium bromide (CTAB)^c^				1%	1%	1%
thymol^b^		2%			2%	
*Lippia origanoides* Kunth essential oil^d^			3%			3%
Milli-Q water^c^	90%	88%	87%	90%	88%	87%

a IUPAC: International Union of Pure and Applied Chemistry.

b Lipid phase.

c Aqueous phase.

d Addition of essential oil.

The NLCs underwent a centrifugation test using a 5430 R centrifuge (Eppendorf, Germany) for 3 cycles of 30 min at 3000 rpm (Brazil, 2014). Following this, the formulations were stored in closed glass containers and maintained at 5 °C (*T*_5°C_), 25 °C (*T*_25°C_), and 37 °C (*T*_37°C_) for 28 days. Samples were analyzed after 24 h (*t*_0_), 7 days (*t*_7_), and 28 days (*t*_28_) postpreparation. The analyses performed included pH measurement (Metrohm model 827, Switzerland), particle size, polydispersity index (PdI), and zeta potential (ZP) using a Zetasizer (Malvern Nano-ZS90, England). Samples were diluted to a 1:500 concentration with Milli-Q water and stirred for 30 min before testing.

The total EO’s load (TL) in the formulations F1_EO_ and F2_EO_, quantified based on the marker compound thymol, was determined using high-performance liquid chromatography (HPLC). The analysis was performed using a Shimadzu (Japan) LC-20A Prominence model coupled with a diode array detector (DAD). The method was executed according to the procedure described by Leal et al. (2003)^39^ and previously validated by our research group.^40^

The EOs’ NLCs were also characterized using transmission electron microscopy (TEM) JEOL (Japan) JEM - 100 CX-II, operated at an acceleration of 80 kV and magnification up to 200,000 times, and employing 1 drop of a 2% (w/v) uranyl acetate solution for negative staining contrast.^41^

### 2.3 Assessment of Minimum Inhibitory Concentration

Fungal sensitivity was evaluated against four yeast fungi - *Candida albicans* (ATCC 64548), *Candida auris* (CDC B11903), *Candida parapsilosis* (ATCC 22019), and *Malassezia furfur* (ATCC 14521) - and three filamentous fungi - *Aspergillus flavus* (ATCC 204304), *Fusarium keratoplasticum* (ATCC 36031), and *Trichophyton rubrum* (ATCC 28188). Both tests were performed following the Clinical Laboratory Standards Institute (CLSI) standard protocol for broth dilution antifungal susceptibility testing of yeasts (M27-A3)^42^ and filamentous fungi (M38-A2).^43^

The assays were conducted using 96-well plates (TPP, Switzerland) and included all NLC formulations within a concentration range of 200–100,000 μg/mL (24.0–12,000 μg/mL–dry basis), using those without active ingredients (F1_B_ and F2_B_) as control formulations. To facilitate comparison and address the limitation of EO’s low solubility in the culture medium, the activity of free EO was evaluated using a mixture containing 1.5% (w/w) EO in a solution of 2% (w/w) dimethyl sulfoxide (DMSO), 20% (w/w) ethanol, and water up to 100% (w/w), homogenized in an ultrasonic bath. The free EO was tested over a concentration range of 1.8 to 940 μg/mL. A similar mixture without the EO was prepared as a solvent control (S) tested in a concentration range of 20–62,500 μg/mL. Voriconazole (Sigma-Aldrich, Germany) served as the antifungal control (C) with a concentration range of 0.0625–32 μg/mL. The studies were performed in duplicate, using 2 rows per fungus for each sample. For the tests, all samples were diluted to 20% with RPMI-1640 (Life Technologies, USA) with l-glutamine (0.3 g/L) buffered to pH 7.0 with MOPS (USB Corporation, USA), supplemented with d-glucose (2.0 g/L). After dilution, 200 μL of the previously diluted samples were added to the first column of wells. Then, 100 μL was removed, transferred to the subsequent well, filled with 100 μL of RPMI, and homogenized with a pipet. After performing the serial dilution, 100 μL of a suspension containing 2.5 × 10^3^ CFU/mL (yeast fungi) or 5 × 10^4^ CFU/mL (filamentous fungi) was added to each well. This procedure was repeated for all fungi. The remaining line wells were negative controls (200 μL of RPMI) and positive controls (100 μL of fungal suspension and 100 μL of RPMI). The plates were placed in an incubator at 37 °C, and readings were taken after 24 and 48 h following CLSI recommendations. The Minimum Inhibitory Concentration (MIC) is the lowest concentration that can completely inhibit fungal growth. One-fold dilution difference was considered significant.

### 2.4 Assessment of Acute Toxicity in *Galleria mellonella* Larvae

The use of *Galleria mellonella* (greater wax moth larvae) in toxicology has gained significant traction as an ethical, cost-effective, and biologically relevant invertebrate model. With physiological and immunological responses comparable to vertebrates, these larvae have demonstrated utility in evaluating the toxicity of various compounds, including antimicrobial agents and nanoparticles, while offering insights into mechanisms such as oxidative stress and immune modulation. Their dose-dependent responses correlate with mammalian models, making them a valuable tool for early stage toxicity assessments and a supplement to in vitro and in vivo systems.^44−48^

The in vivo acute toxicity test was conducted according to the procedures adapted from Gottardo et al. (2019) and Paziani et al. (2019).^49,50^*Galleria mellonella* larvae with 250–350 mg were selected and divided into groups of five larvae injected with the same sample. A total of 19 groups were evaluated simultaneously to investigate the effects of the NLC formulations, the EO itself, and the solvent mixture, while including appropriate controls for comparison. Hence, the experimental design was carefully structured to assess the impact of the formulations’ composition and concentration, the EO’s activity, and the potential influence of the solvents. The NLC formulations (Groups 1–12) included two distinct formulations, F1 and F2, each tested in three versions: blank (B), containing thymol (T), and containing EO. For each version, two concentrations were tested - pure (100%) and diluted (50%, in a 1:2 v/v ratio). This allowed us to evaluate the potential differences in larval tolerance to each formulation and the effects of encapsulated EO. Testing both pure and diluted formulations is common practice, as pure formulations may sometimes cause adverse effects in larvae. The free EO (Groups 13–14) was tested at two concentrations: 5 μL of a 1.5% EO solution and 10 μL of the same solution. These groups enabled a direct comparison of the EO’s effects in its free form against its NLCs’ encapsulated formulations (e.g., F1_EO_ in Groups 5 and 6 and F2_EO_ in Groups 11 and 12). The solvent mixture used to dilute the EO was tested in Groups 15 and 16 at the same volumes as the free EO groups (5 and 10 μL). This was essential to determine whether any observed toxicity could be attributed to the solvents rather than the EO itself. Finally, three control groups (Groups 17–19) were included to ensure reliable interpretation of the results. These comprised a death control group (absolute ethanol injection), a larval inoculation control group (autoclaved water injection), and a larval health control group (no treatment). After injections, the larvae were housed in incubators at 37 °C without food. Evaluations were conducted daily for 5 days, recording the survival and mortality of the larvae. A sample was considered toxic if it resulted in the death of 50% or more of the larvae within this time frame.^51^

### 2.5 Statistics Analysis

The statistical treatment of experimental results was conducted using Prism GraphPad 8.4.3 software (USA), employing appropriate analyses for each type of experiment. The Shapiro-Wilk test was used to assess the normality of populations. For normally distributed data, one-way or two-way ANOVA with Tukey’s post-test was applied, while for non-normally distributed data, the Kruskal–Wallis test with Dunns post-test was used. *p* < 0.05 was considered significant.

## 3 Results

### 3.1 Characterization of *Lippia origanoides* Essential Oil

The GC–MS characterization of EO from *Lippia origanoides* Kunth identified 23 compounds, confirming thymol as the predominant constituent, accounting for 76.06% of the EO’s total composition. The relation of identified components is listed in Table 3.

**Table 3:** Composition of *Lippia origanoides* Essential Oil Determined by Gas Chromatography-Mass Spectrometry According to the Elution Order^a^

compounds	CAS registry number	peak area (%)	IRL
α-thujene	2867-05-2	0.07	923
α-pinene	7785-26-4	0.40	932
β-thujene	3387-41-5	0.05	977
β-myrcene	123-35-3	2.12	987
3-δ-carene	13466-78-9	0.10	1009
δ-carene	29050-33-7	0.61	1016
*p*-cymene	99-87-6	10.67	1023
limonene	5989-54-8	0.38	1027
eucalyptol	470-82-6	0.48	1031
γ-terpinene	99-85-4	1.01	1056
*p*-cymenene	1195-32-0	0.09	1089
α-terpinolene	586-62-9	0.24	1099
umbellulone	24545-81-1	0.24	1168
terpinen-4-ol	562-74-3	0.78	1179
L-α-terpineol	10482-56-1	0.10	1194
thymol methyl ether	1076-56-8	0.76	1227
thymol	89-83-8	76.06	1289
carvacrol	499-75-2	0.25	1296
caryophyllene	87-44-5	3.27	1416
alloaromadendrene	25246-27-9	0.27	1435
β-selinene	17066-67-0	0.11	1452
ledene	21747-46-6	0.10	1487
caryophyllene oxide	1139-30-6	0.72	1577

a Each compound was related to its respective Chemical Abstracts Service (CAS) Registry Number, Peak Area (%), and Linear Retention Indexes (IRL).

### 3.2 Development and Stability Assay of Nanostructured Lipid Carriers

**Figure 1:**
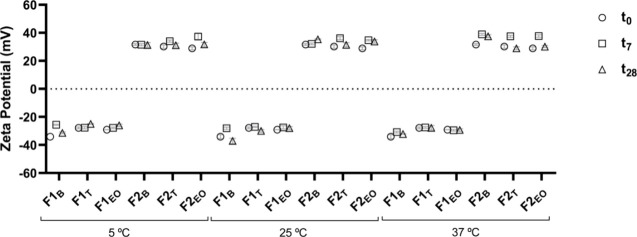
Zeta potential (ZP) of the nanostructured lipid carrier (NLC) formulations in the absence (F1) or presence of the cationic surfactant CTAB (F2) containing thymol (F1_T_ and F2_T_) were analyzed, essential oil (F1_EO_ and F2_EO_), or without any active ingredients (F1_B_ and F2_B_). The formulations were stored at three different temperatures, 5 °C (*T*_5°C_), 25 °C (*T*_25°C_), and 37 °C (*T*_37°C_) and analyzed after 24 h (*t*_0_), 7 days (*t*_7_) and 28 days (*t*_28_) postpreparation.

**Figure 2:**
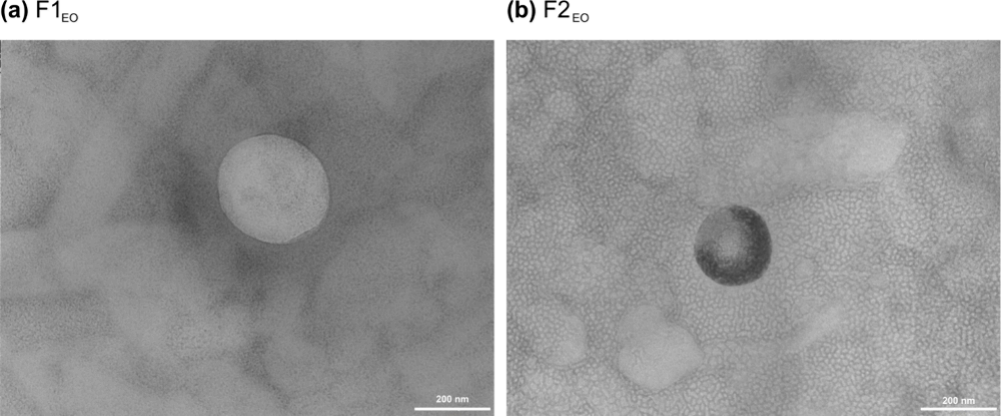
Typical TEM photomicrographs obtained by transmission electron microscopy of nanocarriers formulations F1_EO_ (a) and F2_EO_ (b).

The stability assessment, conducted using the centrifugation method, showed no phase separation in any of the formulations, confirming the developed systems’ stability, as seen in Table 4. The pH measurements of the formulations varied from 4.38 to 5.04 throughout the study period. Regarding particle size (Z-Average), all formulations preserve nanometric dimensions over time. No statistically significant changes (*p* > 0.05) were observed in the particle size between different time points. However, significant variations were recorded for formulation F1_EO_ at 37 °C and F2_T_ at 25 °C (*p* < 0.05). Nevertheless, these variations still remained within the nanometric scale. The analysis of the polydispersity index (PdI) revealed consistent results across different time points and temperatures, indicating high homogeneity and stability of the particles (*p* > 0.05). Most formulations maintained a PdI below 0.2, except F1_B_ and F2_B_, which exhibited a PdI of 0.3. Additionally, as shown in Figure 1, all formulations demonstrated a zeta potential (ZP) around |30| mV, suggesting high electrical stability. The transmission electron micrographs of formulations F1_EO_ (Figure 2a) and F2_EO_ (Figure 2b) indicated predominantly spherical morphology of the nanocarriers and reinforced that they have an average size of 200 nm. The total thymol load (TL) achieved high percentages, with 97.0% in F1_T_, 86.4% in F1_EO_, 98.8% in F2_T_, and 90.3% in F2_EO_, highlighting the effectiveness of the encapsulation methodology used.

**Table 4:** Results of the pH, Particle Size (Z-Average, d.nm) and Polydispersity Index (PdI) of the Nanostructured Lipid Carrier (NLC) Formulations^a^

		pH			Z-average (d.nm)			PdI		
*T* (°C)	NLC	*t*_0_	*t*_7_	*t*_28_	*t*_0_	*t*_7_	*t*_28_	*t*_0_	*t*_7_	*t*_28_
5	F1_B_	4.46 ± 0.03	4.46 ± 0.02	4.56^b^ ± 0.02	253.8 ± 3.0	252.6 ± 3.3	259.2 ± 5.3	0.300 ± 0.049	0.287 ± 0.025	0.295 ± 0.012
	F1_T_	4.65 ± 0.02	4.647 ± 0.01	4.68 ± 0.01	186.3 ± 3.5	184.6 ± 3.3	186.6 ± 2.6	0.175 ± 0.014	0.155 ± 0.045	0.202 ± 0.019
	F1_EO_	4.72 ± 0.01	4.67 ± 0,01	4.69^b^ ± 0.01	176.3 ± 5.0	173.4 ± 4.6	175.9 ± 4.1	0.165 ± 0.017	0.146 ± 0.022	0.141 ± 0.036
	F2_B_	4.93^b^ ± 0.01	4.83 ± 0.01	4.83^b^ ± 0.01	166.9 ± 0.01	162.0 ± 4.3	169.1 ± 2.2	0.284 ± 0.024	0.252 ± 0.018	0.242 ± 0.019
	F2_T_	5.04 ± 0.01	4.99 ± 0.01	5.01^b^ ± 0.01	159.0 ± 2.8	159.3 ± 3.9	157.4 ± 1.4	0.177 ± 0.006	0.158 ± 0.004	0.155 ± 0.028
	F2_EO_	4.87^b^ ± 0.01	4.80 ± 0.01	4.83^b^ ± 0.02	156.5 ± 3.0	157.4 ± 2.9	159.2 ± 1.6	0.186 ± 0.005	0.205 ± 0.023	0.197 ± 0.004
25	F1_B_	4.46 ± 0.02	4.43 ± 0.02	4.40^b^ ± 0.01	253.8 ± 3.0	252.1 ± 1.5	250.9 ± 1.0	0.300 ± 0.049	0.300 ± 0.049	0.284 ± 0.024
	F1_T_	4.65 ± 0.02	4.68 ± 0.02	4.64 ± 0.01	186.3 ± 3.5	182.1 ± 6.3	184.7 ± 1.6	0.175 ± 0.014	0.160 ± 0.020	0.180 ± 0.015
	F1_EO_	4.72 ± 0.01	5.57 ± 0.01	4.38^b^ ± 0.01	176.3 ± 5.1	174.2 ± 6.6	176.9 ± 2.6	0.165 ± 0.017	0.135 ± 0.010	0.150 ± 0.031
	F2_B_	4.93 ± 0.01	4.83 ± 0.01	4.81^b^ ± 0.01	166.9 ± 0.1	164.8 ± 2.5	163.9 ± 3.5	0.284 ± 0.024	0.312 ± 0.012	0.241 ± 0.015
	F2_T_	5.04 ± 0.01	4.96 ± 0.01	4.99 ± 0.01	159.0 ± 2.8	152.8 ± 2.2	152.3^b^ ± 1.5	0.177 ± 0.006	0.147 ± 0.012	0.134 ± 0.015
	F2_EO_	4.87 ± 0.01	4.77 ± 0.01	4.78^b^ ± 0.00	156.5 ± 3.0	156.8 ± 2.2	158.5 ± 1.8	0.186 ± 0.005	0.158 ± 0.010	0.146 ± 0.025
37	F1_B_	4.46 ± 0.03	4.37 ± 0.01	4.21^b^ ± 0.01	253.8 ± 3.0	249.8 ± 1.9	247.8 ± 3.9	0.300 ± 0.049	0.268 ± 0.019	0.299 ± 0.035
	F1_T_	4.65 ± 0.02	4.60 ± 0.01	4.57^b^ ± 0.01	186.3 ± 3.5	183.1 ± 4.1	183.2 ± 2.4	0.175 ± 0.014	0.167 ± 0.003	0.153 ± 0.027
	F1_EO_	4.72 ± 0.01	4.36 ± 0.01	4.23^b^ ± 0.01	176.3 ± 5.1	176.8 ± 3.4	191.9^b^ ± 1.7	0.165 ± 0.017	9.162 ± 0.020	0.208 ± 0.008
	F2_B_	4.93 ± 0.01	4.82 ± 0.01	4.73^b^ ± 0.04	166.9 ± 0.1	165.6 ± 3.3	172.1 ± 4.2	0.284 ± 0.024	0.326 ± 0.048	0.323 ± 0.021
	F2_T_	5.04 ± 0.01	4.93 ± 0.01	4.89^b^ ± 0.01	159.0 ± 2.8	155.2 ± 2.9	159.5 ± 2.4	0.177 ± 0.006	0.170 ± 0.003	0.132 ± 0.014
	F2_EO_	4.87 ± 0.01	4.75 ± 0.00	4.68^b^ ± 0.01	156.5 ± 3.0	156.3 ± 1.7	160.0 ± 0.3	0.186 ± 0.005	0.157 ± 0.026	0.170 ± 0.016

a F1_B_: NLC formulation containing Poloxamer 407, without active ingredients. F1_T_: NLC formulation containing Poloxamer 407, with Thymol. F1_EO_: NLC formulation containing Poloxamer 407, with *Lippia origanoides* essential oil. F2_B_: NLC formulation containing Poloxamer 407 and CTAB, without active ingredients. F2_T_: NLC formulation containing Poloxamer 407 and CTAB, with Thymol. F2_EO_: NLC formulation containing Poloxamer 407 and CTAB, with *Lippia origanoides* essential oil. *T*_5°C_: formulations stored at 5 °C. *T*_25°C_: formulations stored at 25 °C. *T*_37°C_: formulations stored at 37 °C. *t*_0_: analysis performed 24 h postpreparation. *t*_7_: analysis performed 7 days postpreparation. *t*_28_: analysis performed 28 days postpreparation.

b Significant difference between the baseline values (*t*_0_) (*p* < 0.05).

### 3.3 Assessment of Minimum Inhibitory Concentration

The fungal sensitivity assay established the minimum inhibitory concentration (MIC) based on the highest value observed among duplicates. The results are presented in Table 5, expressed in terms of the concentration of NLC formulations (μg/mL) and thymol (μg/mL), based on the chemical composition of the EO determined by GC–MS. The F1_B_ formulation and the solvent control (S) showed no activity against the tested species at the highest concentration used. The Voriconazole control demonstrated values of 0.063 μg/mL for *C. albicans*, *C. auris*, *C. parapsilosis*, and *T. rubrum*; 0.125 μg/mL for *M. furfur*; 0.500 μg/mL for *A. flavus*; and 16.000 μg/mL for *F. keratoplasticum*. To facilitate comparisons between the blank formulations (F1_B_ and F2_B_) and those loaded with thymol or EO, all data have been presented on the same basis (total solids concentration), aligned with the NLC formulations containing thymol or EO.

**Table 5:** Minimum Inhibitory Concentration (MIC) of Nanostructured Lipid Carrier (NLC) Formulations^a^

	F1_B_	F1_T_		F1_EO_		F2_B_	F2_T_		F2_EO_		EO
	NLC (μg/mL)	NLC (μg/mL)	T (μg/mL)	NLC (μg/mL)	T (μg/mL)	NLC (μg/mL)	NLC (μg/mL)	T (μg/mL)	NLC (μg/mL)	T (μg/mL)	T (μg/mL)
*Candida albicans* (ATCC 64548)	N/A	750.0	125.0	125.0	142.6	1500.0	93.6	15.6	46.8	8.9	178.3
*Candida auris* (CDC B11903)	N/A	375.6	62.5	375.6	71.3	N/A	187.2	31.3	375.6	71.3	89.1
*Candida parapsilosis* (ATCC 22019)	N/A	750.0	125.0	750.0	142.6	46.8	24.0	3.9	24.0	4.5	89.1
*Malassezia furfur* (ATCC 14521)	N/A	375.6	62.5	375.6	71.3	6000.0	375.6	62.5	375.6	71.3	89.1
*Aspergillus flavus* (ATCC 2024304)	N/A	376.6	62.5	750.0	142.6	N/A	187.2	31.3	187.2	35.7	178.3
*Fusarium keratoplasticum* (ATCC 36031)	N/A	750.0	750.0	750.0	142.6	N/A	375.6	62.5	375.6	71.3	178.3
*Trichophyton rubrum* (ATCC 28188)	N/A	375.6	62.5	187.2	35.7	780.0	46.8	7.8	46.8	8.9	89.1

a F1_B_: NLC formulation containing Poloxamer 407, without active ingredients. F1_T_: NLC formulation containing Poloxamer 407, with Thymol. F1_EO_: NLC formulation containing Poloxamer 407, with *Lippia origanoides* essential oil. F2_B_: NLC formulation containing Poloxamer 407 and CTAB, without active ingredients. F2_T_: NLC formulation containing Poloxamer 407 and CTAB, with Thymol. F2_EO_: NLC formulation containing Poloxamer 407 and CTAB, with *Lippia origanoides* essential oil. EO: *Lippia origanoides* essential oil. T: Thymol. N/A: No activity at all tested concentrations.

### 3.4 Assessment of Acute Toxicity in *Galleria mellonella* Larvae

**Figure 3:**
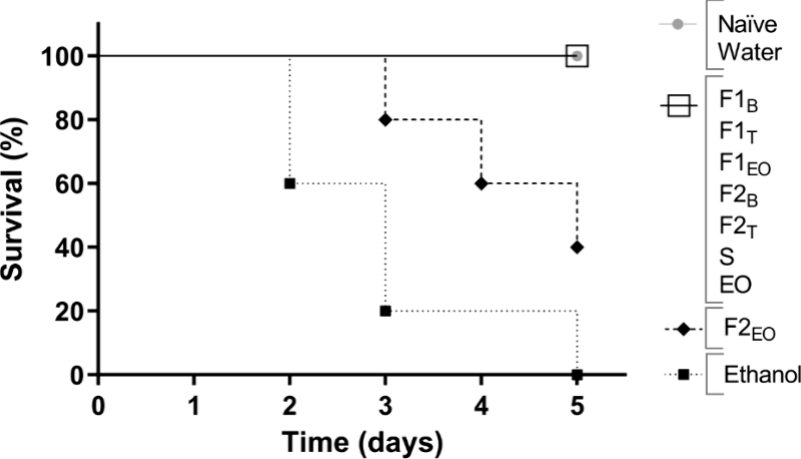
Graph showing the survival rates of *Galleria mellonella* larvae over a five-day time frame. The naïve larvae and those treated with water are indicated by 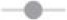 larvae injected with pure formulations F1_B_, F1_T_, F1_EO_, F2_B_, F2_T_, S, EO and are represented by 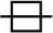; larvae injected with pure formulation F2_EO_ are represented by 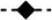; and larvae injected with ethanol are represented by 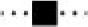.

The toxicity of EO of *Lippia origanoides* encapsulated in NLCs was evaluated using *Galleria mellonella* as an alternative toxicity model. After the 5 days of the experiment, all *G. mellonella* larvae survived to the pure formulations F1_B_, F1_T_, F1_EO_, F2_B_, F2_T_, S, EO, and formulation diluted in water at a 1:2, v/v ratio. Additionally, 40% of the larvae survived with the pure F2_EO_ formulation, and 100% survived the formulation diluted at 1:2. In the control groups, all larvae designated for larval health (naïve) and inoculation tests survived (water), whereas none of the larvae in the control groups meant for mortality testing (ethanol) survived. Thus, it is concluded that the formulations are nontoxic at the tested concentrations (Figure 3).

## 4 Discussion

### 4.1 Characterization of *Lippia origanoides* Essential Oil

GC–MS analysis confirmed thymol as the main component of pepper-rosemary EO, constituting 76.06% of its composition. The high concentration of this monoterpene is particularly beneficial due to its antifungal and anti-inflammatory properties, contributing to the EO’s antifungal activity.^10^ However, when exposed to environmental conditions, pepper-rosemary EO is susceptible to alterations in physicochemical properties and compound volatilization.^12,13^ Its poor aqueous solubility also complicates its incorporation into more complex aqueous-based formulations. Therefore, encapsulation methodologies could protect active ingredients and allow the EO to be applied in suitable pharmaceutical formulations, thus ensuring safe and effective administration.^14,15^

### 4.2 Development and Stability Assay of Nanostructured Lipid Carriers

The stability test indicated that all nanocarriers remained stable throughout the study, with no phase separation or pH changes. Monitoring pH is vital for detecting instability related to hydrolysis of the functional groups of formulation constituents.^52^ Furthermore, maintaining pH within the stratum corneum’s typical range (4.1 to 5.8) is critical to prevent compromising the skin barrier function and causing adverse effects such as burning, itching, redness, or skin desquamation.^38^ Given this, the initial acidic pH (ranging from 2.81 to 2.84) of formulations with CTAB (F2 group) was adjusted using AMP 95. Despite statistically significant pH fluctuations over the 28 days, all NLC formulations remained within a skin-compatible pH, thus rendering them suitable for topical application.

The Z-average, or average hydrodynamic diameter of particles, is a parameter for understanding the behavior of nanocarriers in contact with the human organism.^53^ The developed nanocarriers typically ranged in size from 150 to 190 nm, except for F1_B_, which had a Z-Average of 250 nm. Typically, particles larger than 30 nm are trapped within the stratum corneum and cannot diffuse through the lipid bilayer or permeate the aqueous pores, which is needed to produce systemic effects.^54^ However, the film formation on the stratum corneum surface improves skin hydration by reducing transepidermal water loss, as well the high affinity of NLCs for the lipid bilayer contributes to cell rearrangement, enhancing permeation capacity.^55−58^ According to Souto et al. (2022), the occlusive effect is related to a slight improvement in permeation, and the composition of formulations has the potential to induce alterations in, or potentially compromise, the integrity of the stratum corneum, thereby substantially augmenting absorption.^54^ It is worth noting that the presence of EO did not seem to adversely affect the stability of the formulation since only formulations F1_EO_ (*T*_37°C_) and F2_T_ (*T*_25°C_) showed significant variations between *t*_0_ and *t*_28_. The images obtained by TEM reinforce the Z-Average size range and show a spherical morphology of the nanocarriers.

The particle size distribution directly influences the PdI, presenting low values for uniform distribution.^59^ All formulations exhibit a PdI below 0.3, evidencing a nearly uniform particle size distribution,^60^ with the NLC containing EO tended to exhibit lower PdI. According to Bonilla et al. (2012), pepper-rosemary EO acts positively in emulsification, which might enhance the nanocarriers’ homogeneity and consequently reduce particle size.^61^ This effect is likely due to an optimal balance of the formulation components, particularly the interaction between lipids and surfactants.^54^

ZP reflects the surface charge of nanocarriers, allowing for the monitoring of electrical stability.^62^ All formulations exhibit a ZP of approximately |30| mV, minimizing the occurrence of aggregation or coalescence phenomena and favoring low values of particle size and PdI.^63^ However, the stability mechanisms of nanocarriers result from the combination of electrostatic repulsion and steric hindrance.^64^ Steric stability occurs when the electron clouds of atoms on particle surfaces overlap as two particles come into proximity, whereas electrostatic stability refers to the repulsion between particles carrying electrical charges of the same sign, thereby preventing aggregation.^65^ These mechanisms are employed by the surfactants Poloxamer 407 (nonionic) and CTAB (cationic), respectively.^16^ The formulations in group F1, stabilized by Poloxamer 407, exhibit a negative (−) ZP due to the formation of a polymeric wall at the interface between the lipid nanocarrier and the aqueous medium.^66^ According to Donnelly et al. (1977), the polymer chain end contains a carbanion responsible for the negative surface charge of the NLCs.^67^ However, adding CTAB to the formulations in group F2 promotes a shift in the ZP to positive (+) values. This cationic surfactant promotes an increase in positive charges on the surface of nanocarriers^16^· In both cases, electrostatic stability is achieved.

The assessment of thymol TL by HPLC revealed that all developed nanocarriers retained high concentrations of thymol. The high TL values associated with the stability study suggest that a substantial amount of EO has been incorporated into the NLCs. This substantial load can be attributed to thymol’s lipophilic properties with very low aqueous solubility (∼900 mg/L at 25 °C); therefore, having a stronger affinity with the lipid constituents of the nanocarriers compared to the aqueous medium.^54,68^ Variances emerged between formulations containing EO and isolated thymol, revealing an almost 10% disparity in the TL capacity. Such distinctions may originate from the higher concentration of active compounds in EO-based formulations (3%, w/w) versus those with thymol alone (2%, w/w), potentially leading to competitive loading among other EO constituents. Furthermore, formulations incorporating CTAB exhibited enhanced TL, suggesting that the reduction of particle size and PdI associated with the cationic surfactant may have contributed to the enhancement of thymol retention. Despite fluctuations in TL among the formulations, the abundant presence of this monoterpene considerably contributed to the observed biological activity.

### 4.3 Antifungal Activity

Results of the antifungal activity assays demonstrate the inhibitory activity of pepper-rosemary EO against all tested fungal species, with minimum inhibitory concentration (MIC) values ranging from 117.2 and 234.4 μg/mL, corresponding to concentrations of 89.1 and 178.3 μg/mL of thymol, respectively. EOs with MICs below 500 μg/mL are considered potent antimicrobials.^69^ Therefore, pepper-rosemary essential oil demonstrates potential as an active ingredient against the seven fungal species evaluated in this study.

The antifungal activity of pepper-rosemary EO is attributed to thymol, which, in turn, owes its efficacy to its ability to disrupt the cell membrane of fungi, the structure responsible for regulating the material exchange, stress response, and cell recognition.^70^ In particular, the fungal cell membrane is rich in ergosterol, a sterol component responsible for maintaining cell function, growth, and integrity.^10^ Some authors suggest that thymol can inhibit ergosterol synthesis pathways, leading to reduced ergosterol concentrations in the cell membrane and the accumulation of methyl valerate, which compromises both structural and metabolic integrity, ultimately resulting in cell death.^9,71^ Thymol induces the repression of three genes that code for oxysterol-binding proteins necessary for ergosterol biosynthesis, blocking the production of this sterol.^72^ However, other authors suggest that the action of thymol is dependent on the hydrophobicity of the molecule and its ability to form hydrogen bonding, allowing it to interact with the cell membrane and causing damage to the structure and permeability of the fungal cell, leading to irreversible damage and the leakage of intracellular content.^73^

This strategy of compromising the cell membrane corresponds to the mechanism of action of many traditional antifungals. The class of azole antifungals inhibits the ergosterol biosynthesis pathway at converting lanosterol to demethyl-lanosterol.^74^ Voriconazole, the antifungal adopted as a control, is a second-generation triazole agent with high in vitro and in vivo activity against various yeast and filamentous fungi that act as human pathogens.^75,76^ Although it is a derivative of Fluconazole, its spectrum of action is broadened, and its activity is enhanced.^76^ Thus, following the parameters pre-established by CLSI protocols, Voriconazole was active against all the fungal species evaluated in this study, with most species requiring concentrations of less than 0.5 μg/mL, except for *F. keratoplasticum*, which required a MIC of 16 μg/mL.

Concerning the nanocarriers in the F1 group, the F1_B_ formulation could not inhibit fungal growth at any of the concentrations evaluated. In contrast, the formulations containing thymol (F1_T_ and F1_EO_) were active against all seven fungal species. The MIC values for the two formulations showed no differences (≤1 MIC), reinforcing that the antifungal activity is linked to the presence of the monoterpene thymol. In comparison with the performance of free EO, no differences (≤1 MIC) in antifungal activity were observed.

On the other hand, the formulation of group F2 in the absence of an active ingredient (F2_B_) proved to be active against *C. albicans*, *C. parapsilosis*, *M. furfur*, and *T. rubrum* due to the presence of the cationic surfactant, CTAB. Fungal cells have a net negative charge and, therefore, have a high affinity for the positive charge of cationic surfactants.^77^ As a result, they can interact with the negatively charged surface of the cells, promoting a change in the surface charge and preventing the interaction of fungal cells with host tissue and clinically implanted devices.^5,77^ CTAB is a quaternary ammonium surfactant capable of reversing the fungal cell’s surface charge from negative to positive, without promoting cell lysis.^77,78^ Similarly, positively charged nanocarriers demonstrate a strong affinity for fungal cells.^79^ The correlation of this property with the lipid composition of NLCs may enhance the disruption of cell walls, facilitating membrane permeation.

*Candida auris*, *A. flavus*, and *F. keratoplasticum* did not have their growth inhibited by F2_B_, suggesting a lower sensitivity to CTAB. The lower sensitivity of the fungi *A. flavus* and *F. keratoplasticum* to the CTAB surfactant may be associated with the composition of the cell wall of filamentous fungi. While yeasts have only 2% of their dry weight composed of chitin, filamentous fungi have 10 to 15% of their mass corresponding to fiber, contributing to greater rigidity and cell wall resistance.^80^ Although also considered a filamentous fungus, *T. rubrum* is an obligate pathogenic species, while *A. flavus* and *F. keratoplasticum* are environmental fungi and opportunistic pathogens.^24,28,31^ The exposure of environmental fungi to stressful conditions can favor the selection of resistant species with more complex virulence mechanisms than fungi that exclusively infect animals and humans.^81^ For example, the antifungals used in agriculture contribute to cross-resistance phenomena with clinical antifungals, making it difficult to treat infected individuals.^82^

*C. auris*, despite being a yeast fungus, is considered a multidrug-resistant species whose isolates have shown resistance to all four classes of human antifungal drugs.^5^ Although these mechanisms are not yet fully understood, they can minimize the contact of CTAB with the cell wall and consequently decrease the fungus’ sensitivity to the substance.^5^ Considering that *Candida* spp. has a high capacity for producing biofilms, one of the possibilities would be that these three-dimensional structures have provided the yeast with protection against the antimicrobial.^5,10,83^ It is worth noting that although the other *Candida* spp. can also produce biofilm, the strain of *C. auris* (CDC B11903) used in the study is the only one with multidrug resistance. Literature reports showed several reference strains of *C. albicans* produced less biofilm than *C. auris* (CDC B11903).^83,84^

Another possible explanation for the superior resistance of *C. auris* to CTAB is related to a low concentration of negative charges in the cell surface and, consequently, minor interaction with the cationic surfactant. The availability of negatively charged groups on the cell surface varies between the different *Candida* spp.^85^ Given this, studies aimed at evaluating the ionic interaction between chitosan and *Candida* spp., pointed out that *C. parapsilosis* has a higher concentration of negative charges than *C. albicans* and, consequently, a greater affinity for positively charged compounds, being more sensitive to chitosan.^85^ Similarly, *C. parapsilosis* proved to be significantly (>1 MIC) more sensitive to the formulations in group F2 than *C. albicans*. Although these studies did not evaluate the surface charge of *C. auris*, it is possible to assume that a lower availability of negatively charged groups is responsible for its lower sensitivity to CTAB, which exhibited higher MIC values than pure EO and NLC formulations.

Unlike F2_B_, the formulations F2_T_ and F2_EO_ were active against all species, so those not sensitive to F2_B_ had their growth inhibited. As a result of the absence of or low sensitivity to CTAB, the formulations in groups F1 and F2, as well as the free EO, did not show significant differences (≤1 MIC) in their activity against *C. auris*, *A. flavus*, and *F. keratoplasticum*. It is worth noting that, unlike CTAB, thymol can inhibit biofilm production by inhibiting the metabolism of fatty acids, contributing to an increase in the concentration of reactive oxygen species (ROS) and, consequently, the state of oxidative stress.^10^ Thus, even if *C. auris* presents biofilm production as a resistance mechanism, the activity of EO is not compromised.

The species sensitive to F2_B_, particularly *C. albicans*, *C. parapsilosis*, and *T. rubrum*, showed a significant improvement (>1 MIC) in antifungal activity compared to the F1 group. The F2 formulations, particularly F2_T_ and F2_EO_, demonstrated substantially lower MICs compared to free EO, emphasizing the enhanced antifungal efficacy. This improvement may be attributed to the synergistic mechanisms of action of thymol and CTAB. The comparison between F2_B_ and F2_T_/F2_EO_ indicates that while CTAB positively influences activity (>1 MIC), the presence of thymol enhances the antifungal effect, demonstrating its primary contribution to the observed activity of the NLCs.

### 4.4 Assessment of Acute Toxicity in *Galleria mellonella* Larvae

Tests on *Galleria mellonella* larvae can predict acute toxicity in mammals ethically and cost-effectively.^86^*G. mellonella* larvae possess an immune system that is both structurally and functionally similar to the innate immune system found in mammals, which aids in evaluating the efficacy and pharmacokinetic behavior of antimicrobials.^51,87^ The results of the NLCs’ toxicity suggest that the NLCs developed do not promote acute toxicity in dilutions of 50% (v/v) or lower. EO in the concentrations of 1.5 and 3% has also proven safe. Moreover, except for formulation F2_EO_, all the formulations proved safe even when administered in pure form. These findings suggest that the observed toxicity of F2_EO_ stems from the combination of CTAB and the pepper-rosemary EO. However, additional studies are required to affirm the formulation’s toxicity and identify the specific substance accountable for the adverse effect, especially considering other formulations containing these components did not exhibit any toxicity. Hence, although CTAB is known to induce skin irritation, leading to restrictions on its use in topical products, the presence of CTAB alone was not decisive for the toxic effect, especially after diluting the formulations.^7^ The correction of acidic pH may have contributed to an improvement in the safety profile of CTAB, justifying the results obtained in the conducted toxicity test. Despite being known as a toxic compound, CTAB remains widely used in the development of nanocarriers due to its low cost and ability to minimize aggregation phenomena.^7^

These findings reinforce that *L. sidoides*’ EO and the nanocarriers developed constitute a promising alternative to traditional antifungals, results that agree with the literature. Further studies are needed to determine the minimum fungicidal concentrations (MFC) and tests on different strains, toxicity, and cell permeability studies to understand the spectrum of action of the formulations and assess the potential for in vivo studies.

## 5 Conclusions

The developed nanocarriers demonstrated stability and suitability for the proposed topical application. The MIC results highlighted the antifungal potential of both free and nanoencapsulated EOs against the seven evaluated fungal species, even at reduced concentrations. Additionally, acute toxicity assessments in *Galleria mellonella* larvae indicated no toxicity of the NLCs at concentrations of 50% or lower. This study elucidated the differential fungal responses to EO and the cationic surfactant CTAB, suggesting a supporting role for CTAB in the observed antifungal activity of NLCs but not solely responsible for it. Specifically, *Candida albicans*, *Candida parapsilosis*, *Malassezia furfur*, and *Trichophyton rubrum* exhibited sensitivity to CTAB, resulting in enhanced antifungal activity in formulations from groups F1 and F2. Conversely, *Candida auris*, *Aspergillus flavus*, and *Fusarium keratoplasticum* showed no sensitivity to CTAB, making formulations from group F1 more advantageous for these species. Furthermore, this study contributes to developing high-value products that promote Brazilian biodiversity and stimulate wealth generation for local communities.

## Supporting Information Available

Primary Mass Spectrometry Data from GC–MS Analysis (Shimadzu GCMS QP-2010, Japan) of *Lippia origanoides* Kunth Essential Oil; mass spectrometry graphs of molecular and product ions associated with the peaks identified in the GC–MS analysis of *Lippia origanoides* Kunth essential oil; each figure corresponds to a specific peak, detailing the identified compound, retention time, and product ions, as reported by Adams (2007); chemical composition of *Lippia origanoides* Kunth Essential Oil obtained from GC–MS Analysis (Shimadzu GCMS QP-2010, Japan) using Homologous Alkanes (C_8_–C_20_) for Constituent Identification as reported by Adams (2007), the number of peaks, retention time (R. Time), peak area, compound name, and mass-to-charge ratio molecular ion (Base *m*/*z*) available; calculation of Kováts and Linear Retention Indices (IRL) from Primary GC–MS Data (Shimadzu GCMS QP-2010, Japan) of *Lippia origanoides* Kunth Essential Oil Using Homologous Alkanes (C_8_–C_20_) Based on Adams (2007); and peaks 1–4 correspond to the detection of the solvents used in the method and do not have corresponding values in the alkane series, preventing IRL determination (PDF)
