# Supplementary material for: Surfactant-Driven Effects on the Antifungal Activity of Lippia origanoides Kunth Essential Oil Encapsulated in Lipid-Based Nanosystems
Source: ACS Omega. 2025 Feb 20;10(8):7876–87. doi: 10.1021/acsomega.4c08578 (PMC11886426; doi:10.1021/acsomega.4c08578)
Supplement: Supplementary file 1 — ao4c08578_si_001.pdf [file ao4c08578_si_001.pdf]

SUPPORTING INFORMATION

Surfactant-Driven Effects on the Antifungal Activity of *Lippia origanoides* Kunth Essential Oil Encapsulated in Lipid-Based Nanosystems

Gabriela Alberto Gil[a], Letícia Kakuda[a], Ludmilla Tonani[a], Marcia Regina von Zeska Kress[a], Wanderley Pereira Oliveira[a]*

[a] University of São Paulo, School of Pharmaceutical Sciences of Ribeirão Preto, 14040-903, Ribeirão Preto, Brazil

*Corresponding author: wpoliv@usp.br, ORCID: 0000-0003-4356-2508

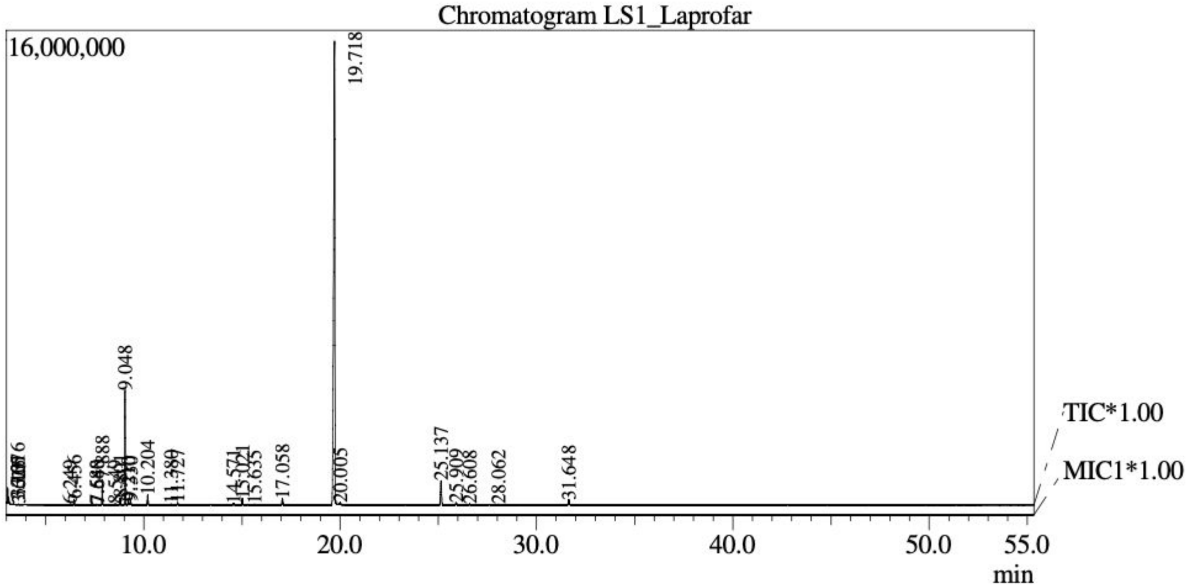

Figure S1. Primary Mass Spectrometry Data from GC-MS Analysis (Shimadzu GCMS QP-2010, Japan) of *Lippia origanoides* Kunth Essential Oil.

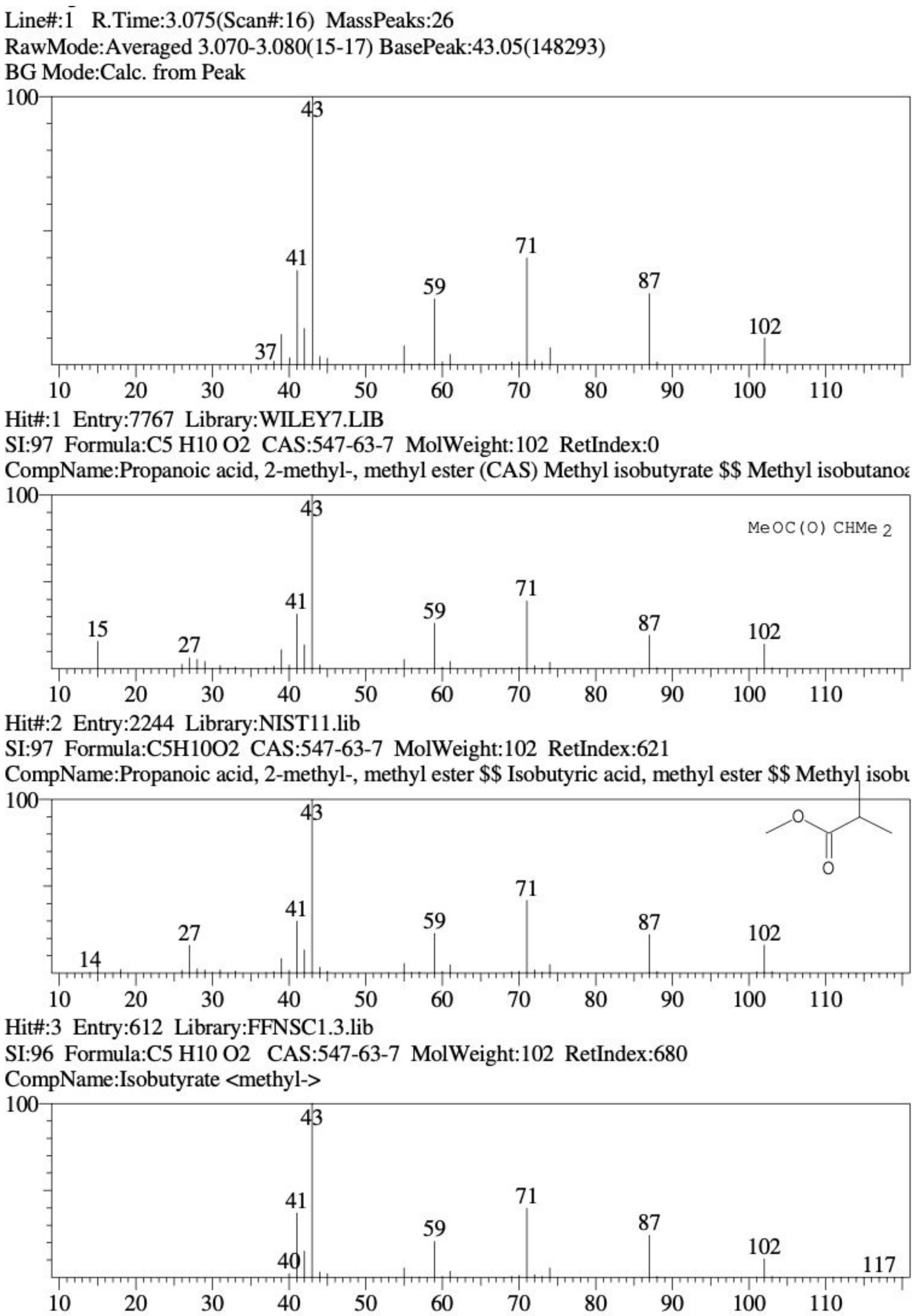

Figure S2. Mass spectrometry graphs of the molecular ion associated with Peak 1, corresponding to compound Propanoic acid, 2-methyl-, methyl ester (CAS) Methyl isobutyrate reported by Adams (2007), with a retention time of 3.075 min, as well as the product ions (Hit#:1, Hit#:2, and Hit#:3).

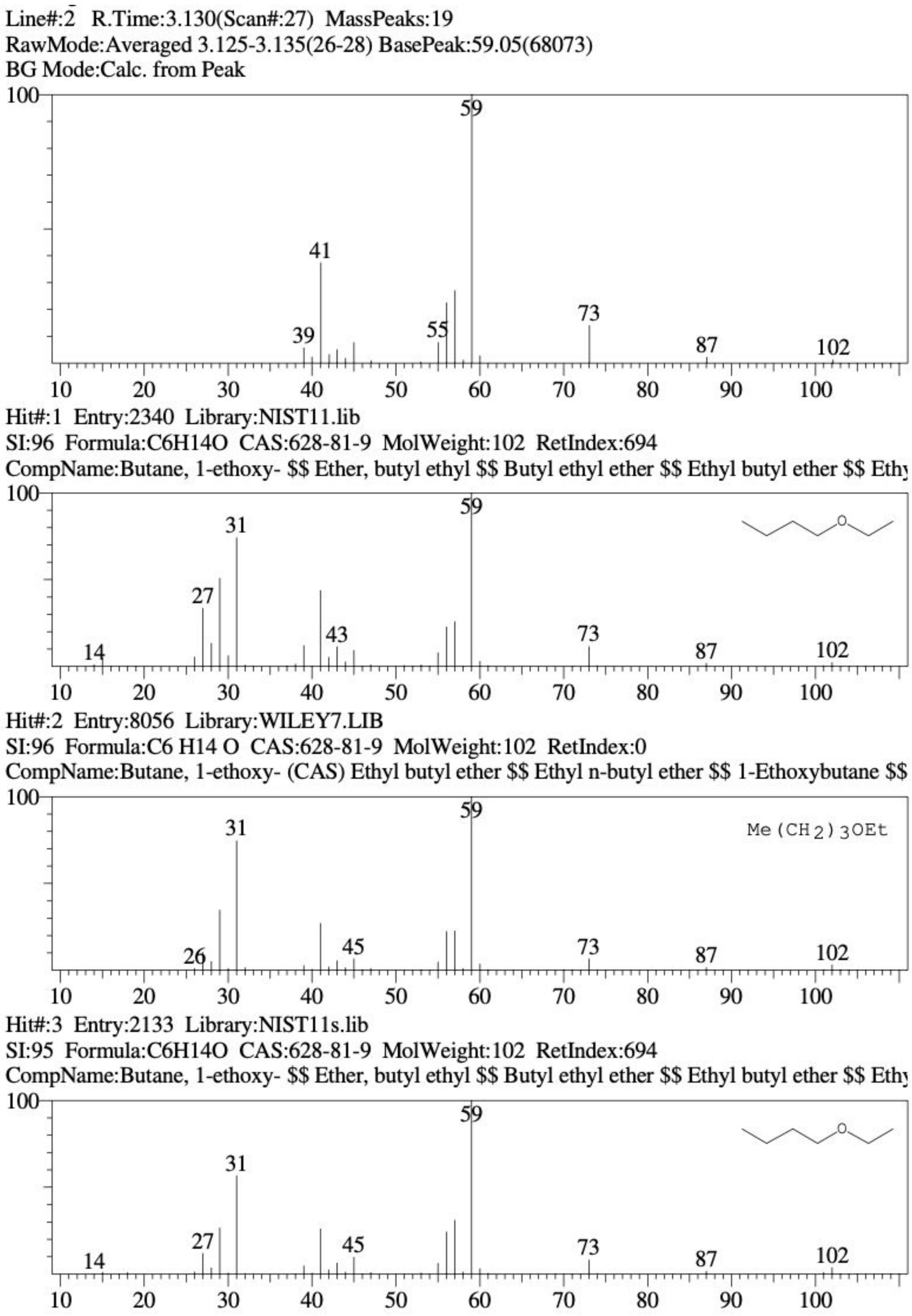

Figure S3. Mass spectrometry figure of the molecular ion associated with Peak 2, corresponding to compound Butane, 1-ethoxy- reported by Adams (2007), with a retention time of 3.130 min, as well as the product ions (Hit#:1, Hit#:2, and Hit#:3).

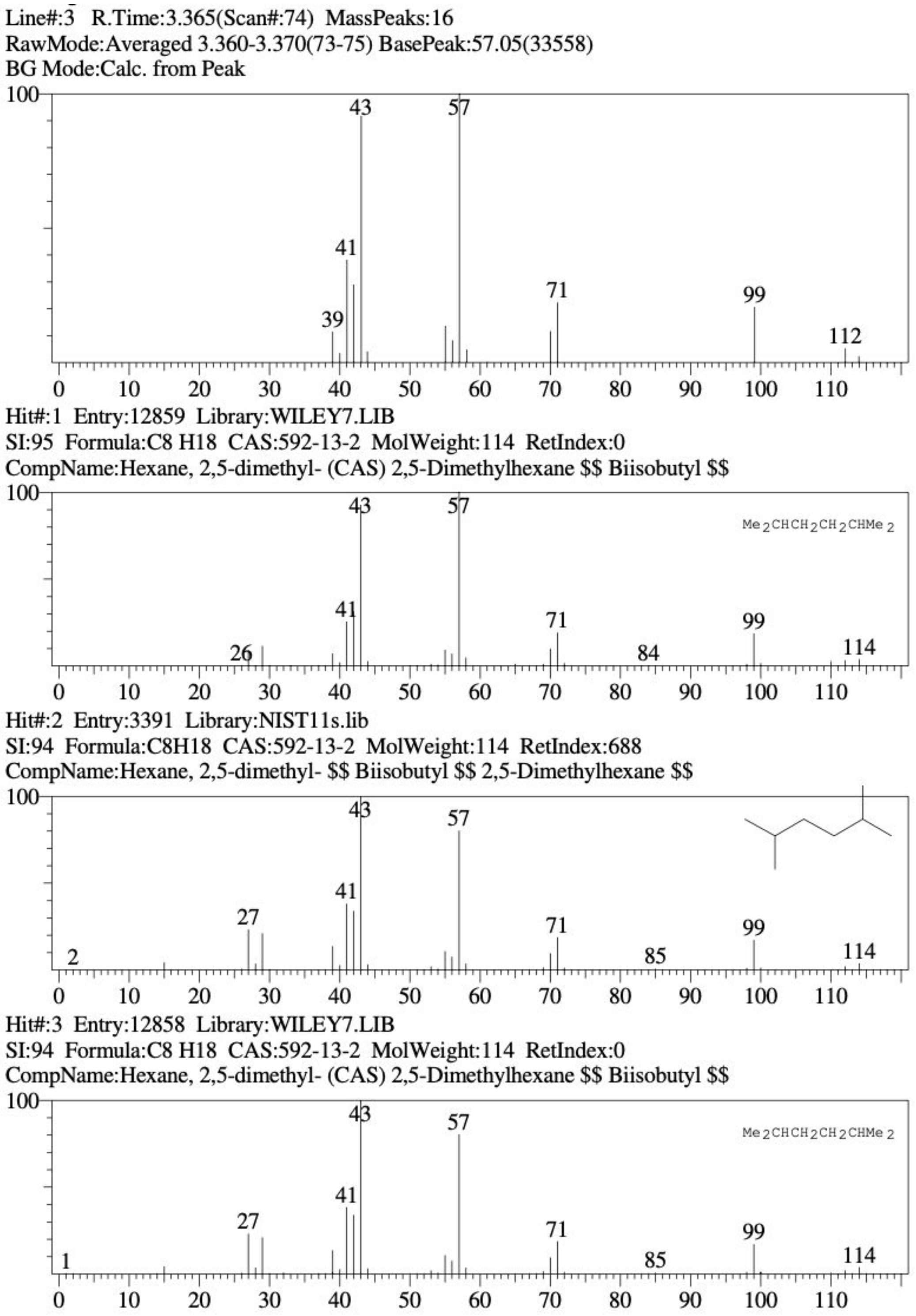

Figure S4. Mass spectrometry figure of the molecular ion associated with Peak 3, corresponding to compound Hexane, 2,5-dimethyl- (CAS) 2,5-Dimethylhexane reported by Adams (2007), with a retention time of 3.365 min, as well as the product ions (Hit#:1, Hit#:2, and Hit#:3).

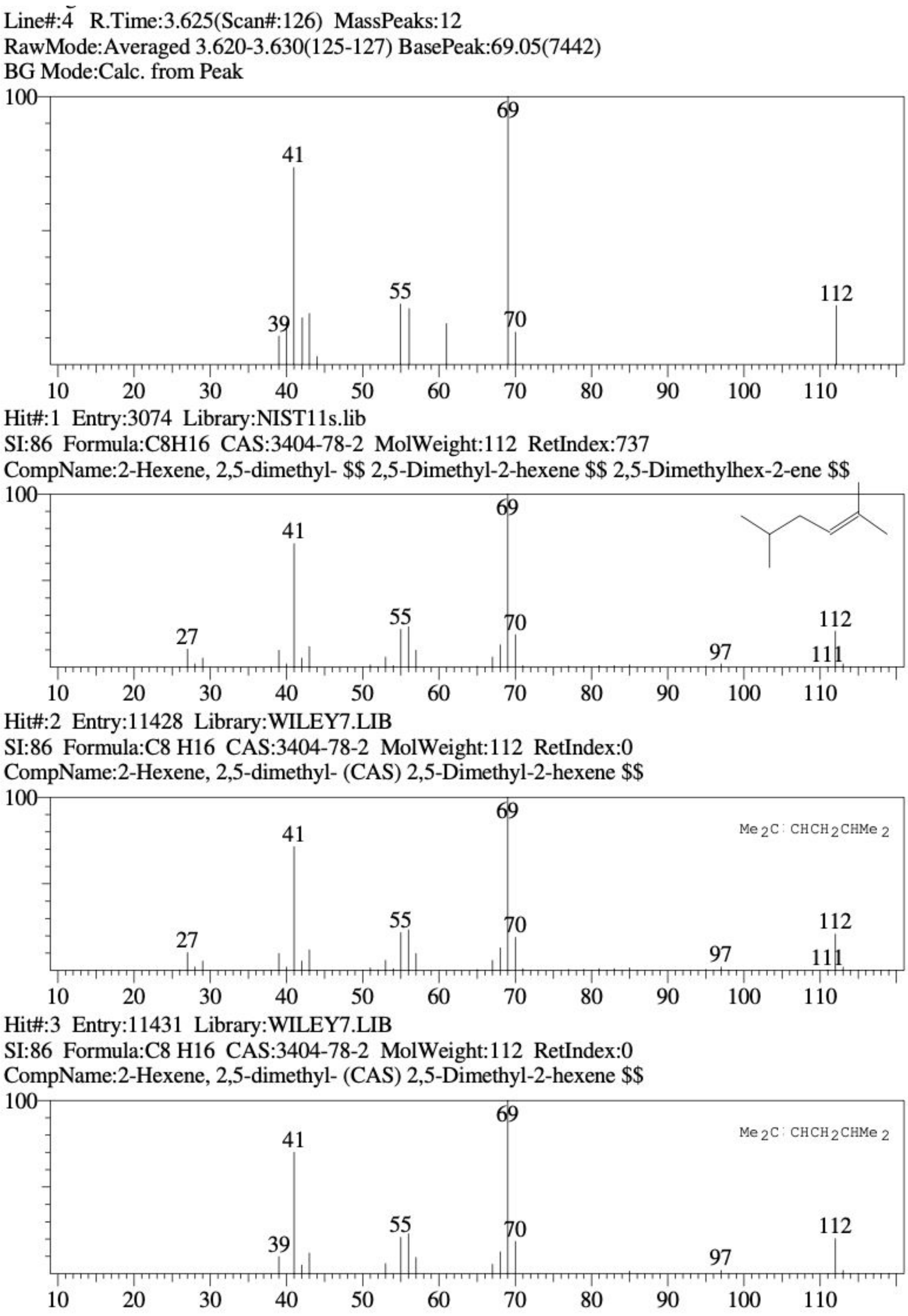

Figure S5. Mass spectrometry figure of the molecular ion associated with Peak 4, corresponding to compound 2-Hexene, 2,5-dimethyl- reported by Adams (2007), with a retention time of 3.625 min, as well as the product ions (Hit#:1, Hit#:2, and Hit#:3).

Line#:5 R.Time:6.250(Scan#:651) MassPeaks:13
RawMode:Averaged 6.245-6.255(650-652) BasePeak:93.05(10953)
BG Mode:Calc. from Peak

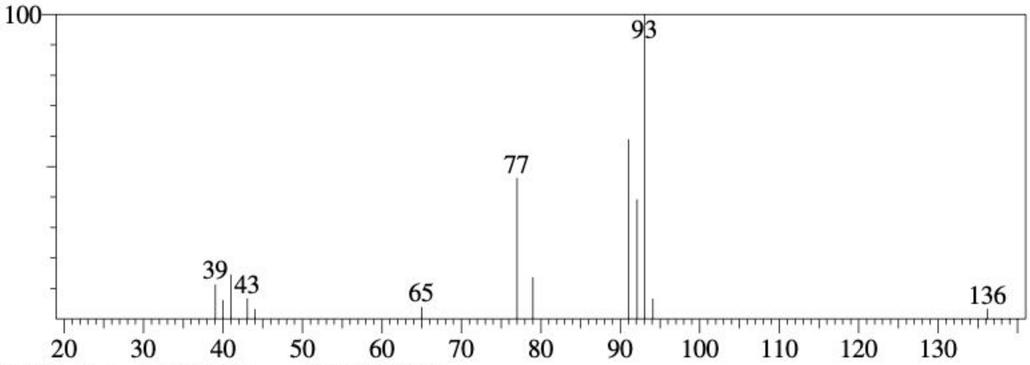

Hit#:1 Entry:26420 Library:WILEY7.LIB
SI:94 Formula:C10 H16 CAS:2867-05-2 MolWeight:136 RetIndex:0
CompName:.alpha.-Thujene $$ Bicyclo[3.1.0]hex-2-ene, 2-methyl-5-(1-methylethyl)- (CAS) Origan

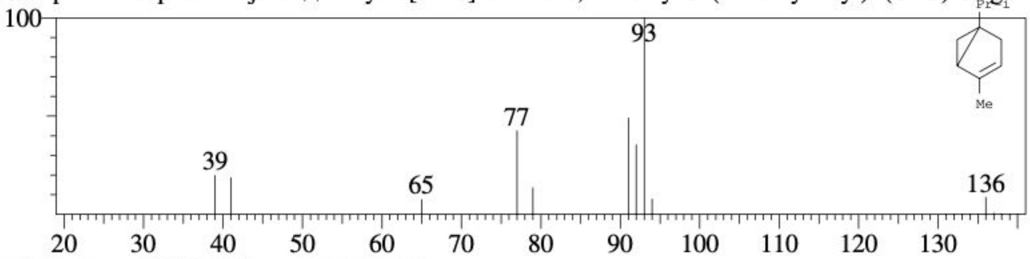

Hit#:2 Entry:26260 Library:WILEY7.LIB
SI:91 Formula:C10 H16 CAS:99-83-2 MolWeight:136 RetIndex:0
CompName:l-Phellandrene $$ 1,3-Cyclohexadiene, 2-methyl-5-(1-methylethyl)- (CAS) p-Mentha-1,

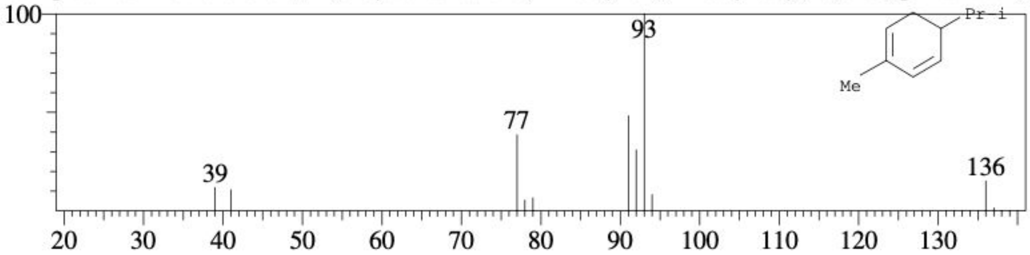

Hit#:3 Entry:26414 Library:WILEY7.LIB
SI:90 Formula:C10 H16 CAS:2867-05-2 MolWeight:136 RetIndex:0
CompName:.alpha.-Thujene $$ Bicyclo[3.1.0]hex-2-ene, 2-methyl-5-(1-methylethyl)- (CAS) Origan

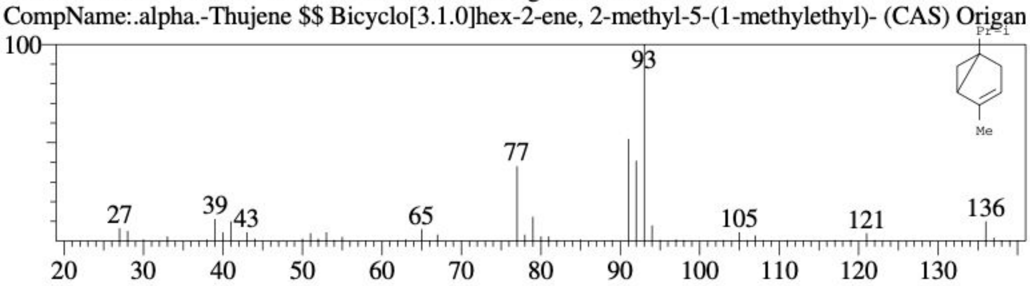

Figure S6. Mass spectrometry figure of the molecular ion associated with Peak 5, corresponding to compound alpha-Thujene reported by Adams (2007), with a retention time of 6.250min, as well as the product ions (Hit#:1, Hit#:2, and Hit#:3).

Line#:6 R.Time:6.455(Scan#:692) MassPeaks:28
RawMode:Averaged 6.450-6.460(691-693) BasePeak:93.05(46753)
BG Mode:Calc. from Peak

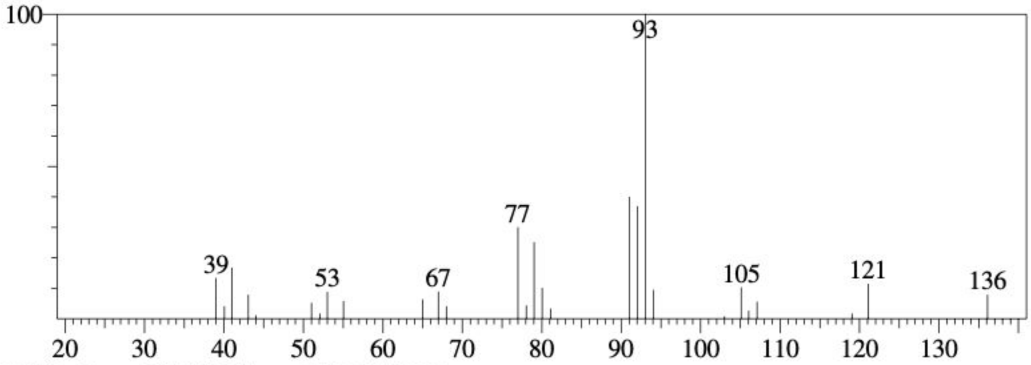

Hit#:1 Entry:26447 Library:WILEY7.LIB
SI:96 Formula:C10 H16 CAS:80-56-8 MolWeight:136 RetIndex:0
CompName:.ALPHA.-PINENE, (-)- $$ Bicyclo[3.1.1]hept-2-ene, 2,6,6-trimethyl- (CAS) Pinene $$

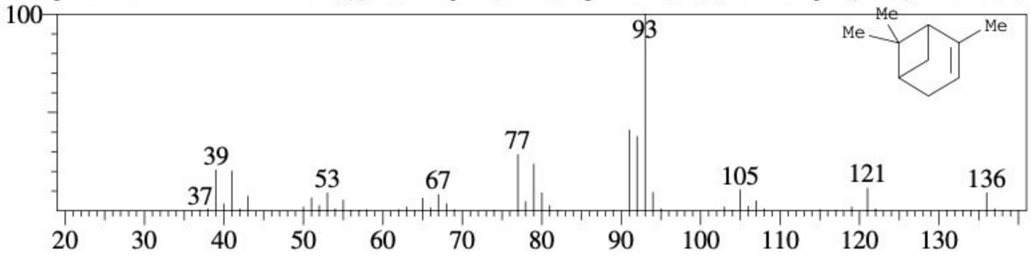

Hit#:2 Entry:26444 Library:WILEY7.LIB
SI:96 Formula:C10 H16 CAS:80-56-8 MolWeight:136 RetIndex:0
CompName:.ALPHA.-PINENE, (-)- $$ Bicyclo[3.1.1]hept-2-ene, 2,6,6-trimethyl- (CAS) Pinene $$

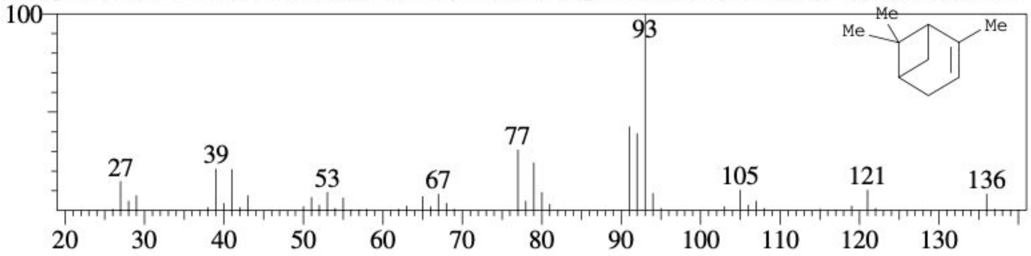

Hit#:3 Entry:6669 Library:NIST11s.lib
SI:95 Formula:C10H16 CAS:80-56-8 MolWeight:136 RetIndex:948
CompName:.alpha.-Pinene $$ Bicyclo[3.1.1]hept-2-ene, 2,6,6-trimethyl- $$ 2-Pinene $$ 2,6,6-Trime

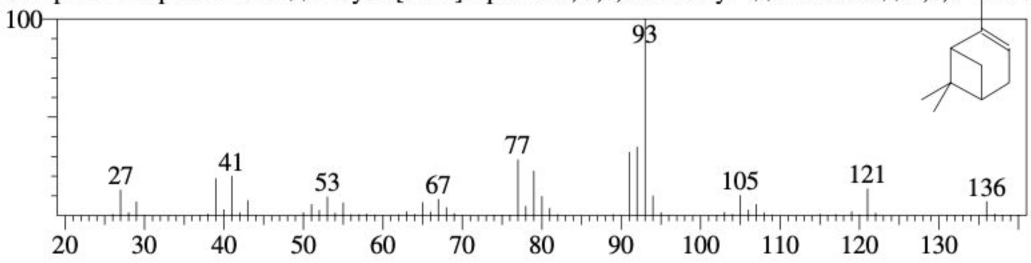

Figure S7. Mass spectrometry figure of the molecular ion associated with Peak 6, corresponding to compound Alpha-Pinene, (-)- reported by Adams (2007), with a retention time of 6.455min, as well as the product ions (Hit#:1, Hit#:2, and Hit#:3).

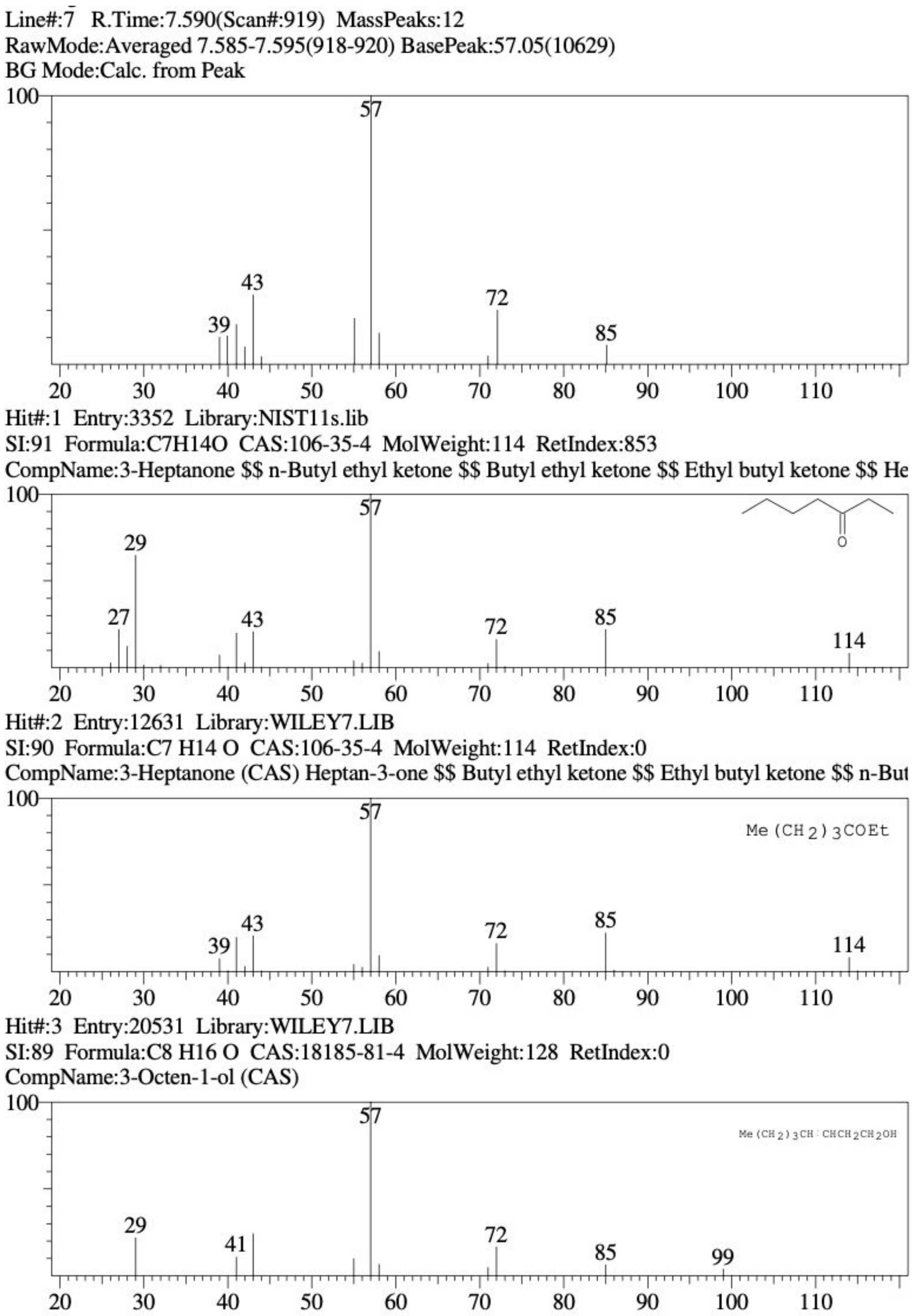

Figure S8. Mass spectrometry figure of the molecular ion associated with Peak 7, corresponding to compound 3-Heptanone reported by Adams (2007), with a retention time of 7.590min, as well as the product ions (Hit#:1, Hit#:2, and Hit#:3).

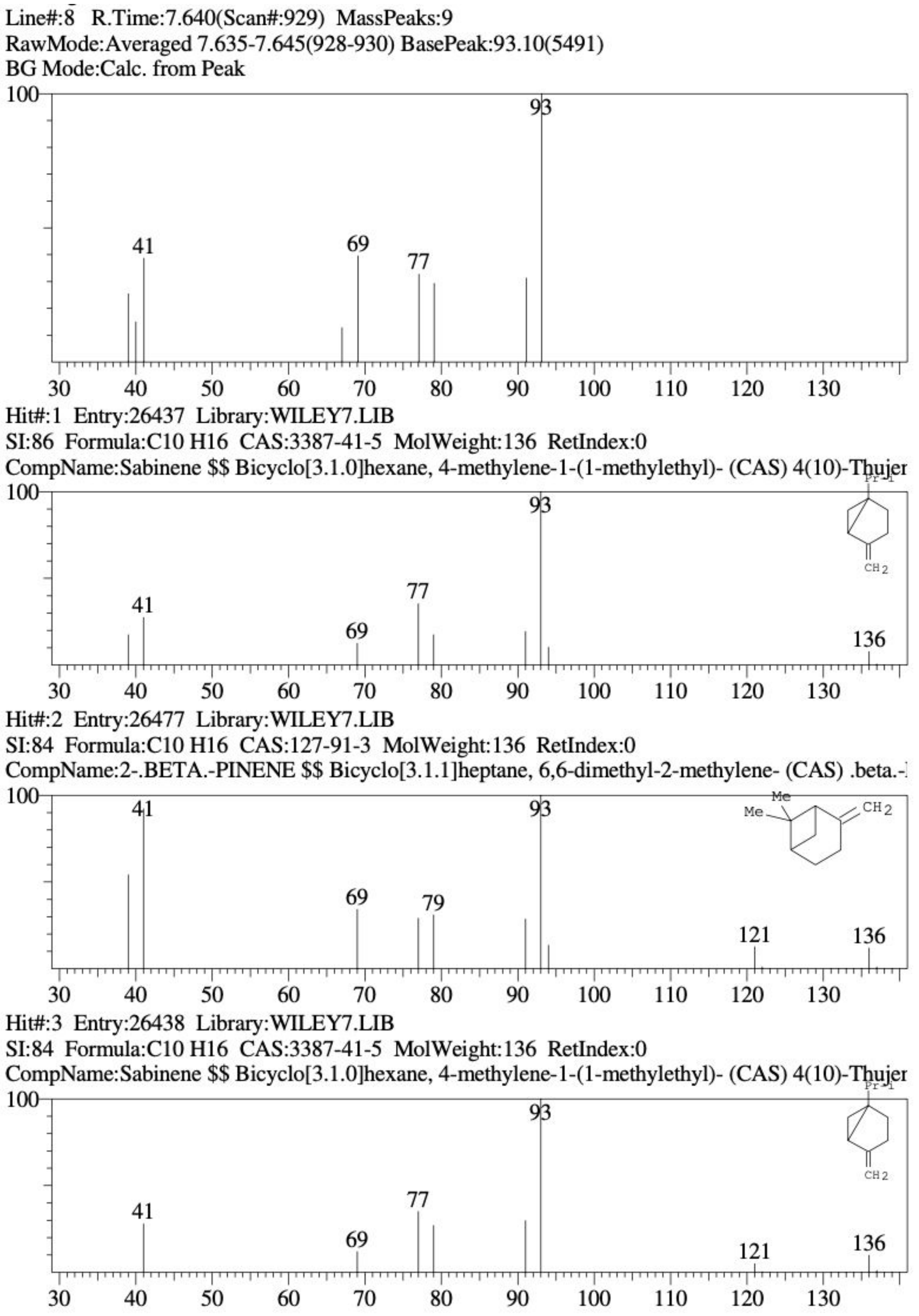

Figure S9. Mass spectrometry figure of the molecular ion associated with Peak 8, corresponding to compound Sabinene reported by Adams (2007), with a retention time of 7.640min, as well as the product ions (Hit#:1, Hit#:2, and Hit#:3).

Line#:9 R.Time:7.890(Scan#:979) MassPeaks:38
RawMode:Averaged 7.885-7.895(978-980) BasePeak:41.00(182363)
BG Mode:Calc. from Peak

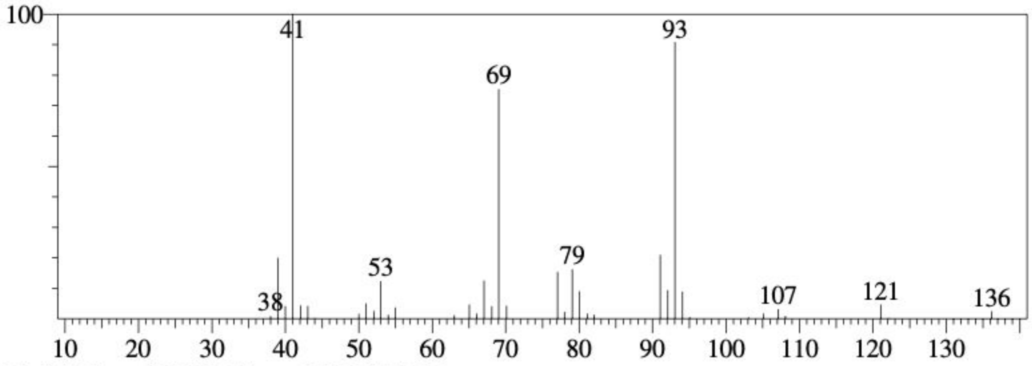

Hit#:1 Entry:26199 Library:WILEY7.LIB
SI:96 Formula:C10 H16 CAS:123-35-3 MolWeight:136 RetIndex:0
CompName:.beta.-Myrcene $$ 1,6-Octadiene, 7-methyl-3-methylene- (CAS) 2-Methyl-6-methylene-

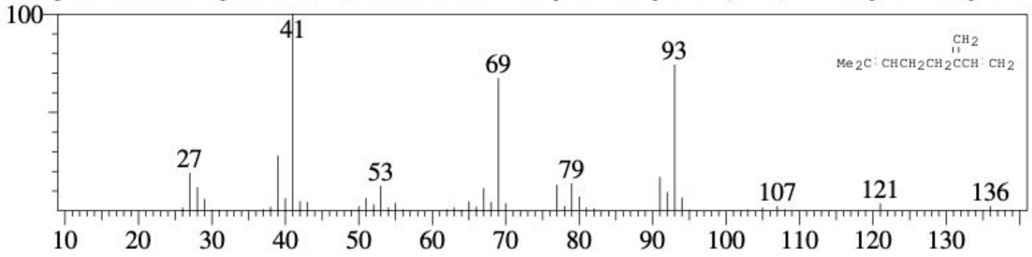

Hit#:2 Entry:6606 Library:NIST11s.lib
SI:96 Formula:C10H16 CAS:123-35-3 MolWeight:136 RetIndex:958
CompName:.beta.-Myrcene $$ 1,6-Octadiene, 7-methyl-3-methylene- $$ Myrcene $$ 7-Methyl-3-m

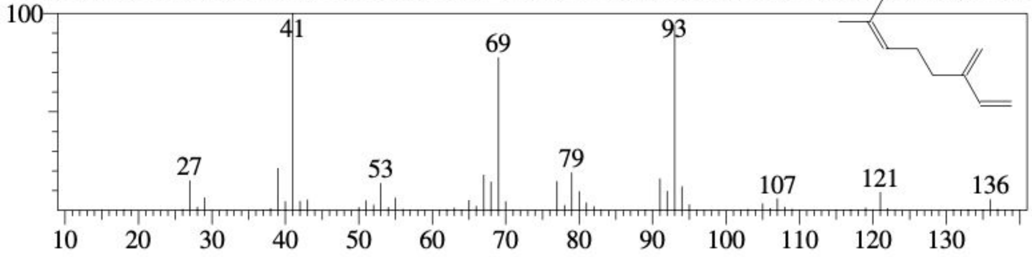

Hit#:3 Entry:628 Library:FFNSC1.3.lib
SI:96 Formula:C10 H16 CAS:123-35-3 MolWeight:136 RetIndex:991
CompName:Myrcene

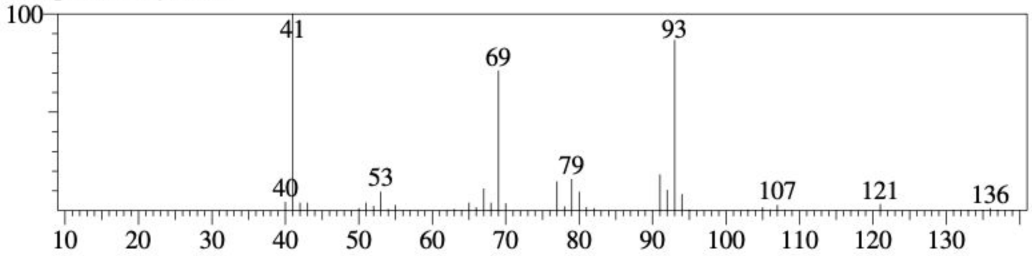

Figure S10. Mass spectrometry figure of the molecular ion associated with Peak 9, corresponding to compound beta-Myrcene reported by Adams (2007), with a retention time of 7.890min, as well as the product ions (Hit#:1, Hit#:2, and Hit#:3).

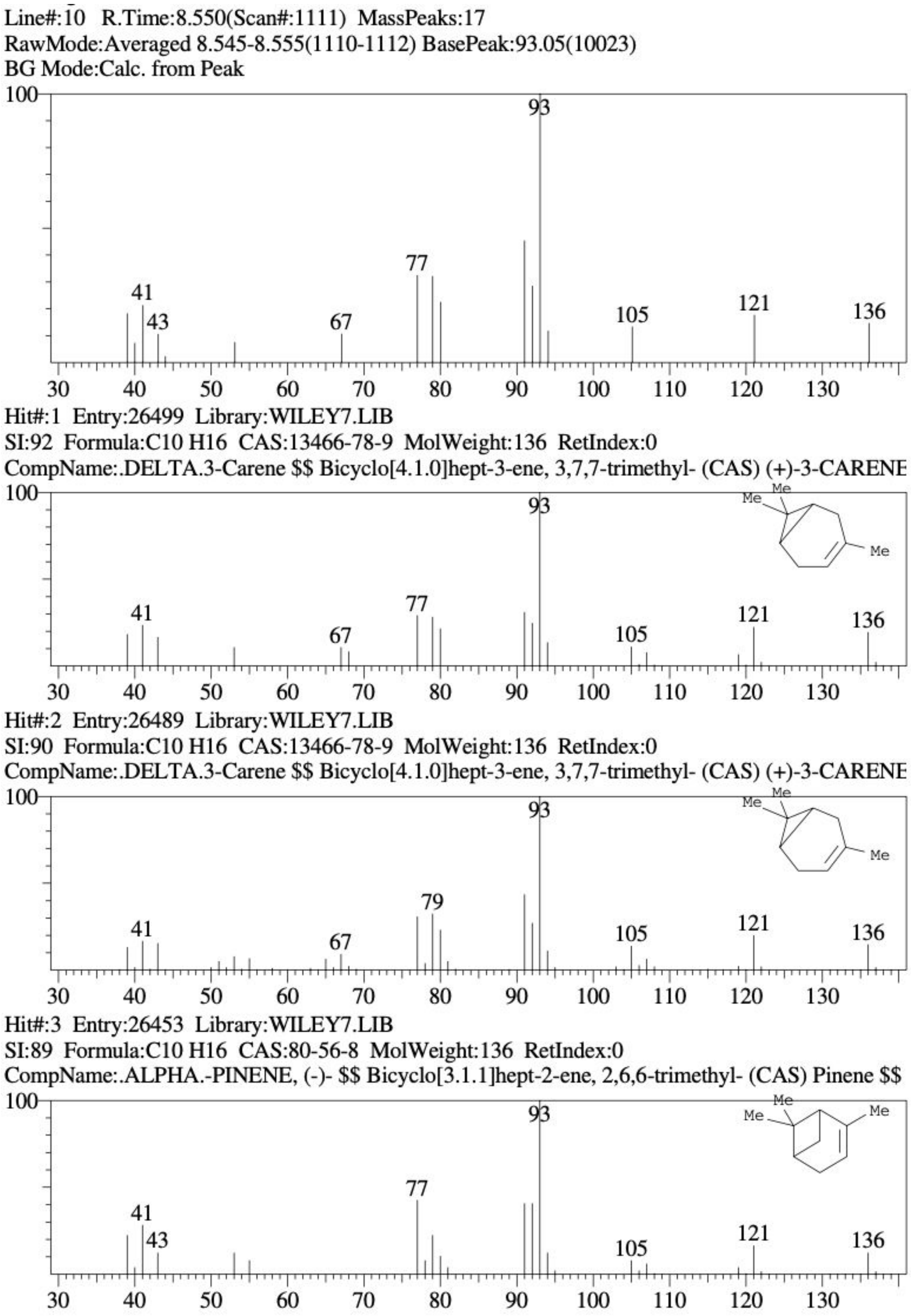

Figure S11. Mass spectrometry figure of the molecular ion associated with Peak 10, corresponding to compound Delta.3-Carene reported by Adams (2007), with a retention time of 8.550min, as well as the product ions (Hit#:1, Hit#:2, and Hit#:3).

Line#:11 R.Time:8.800(Scan#:1161) MassPeaks:33
RawMode:Averaged 8.795-8.805(1160-1162) BasePeak:121.10(40457)
BG Mode:Calc. from Peak

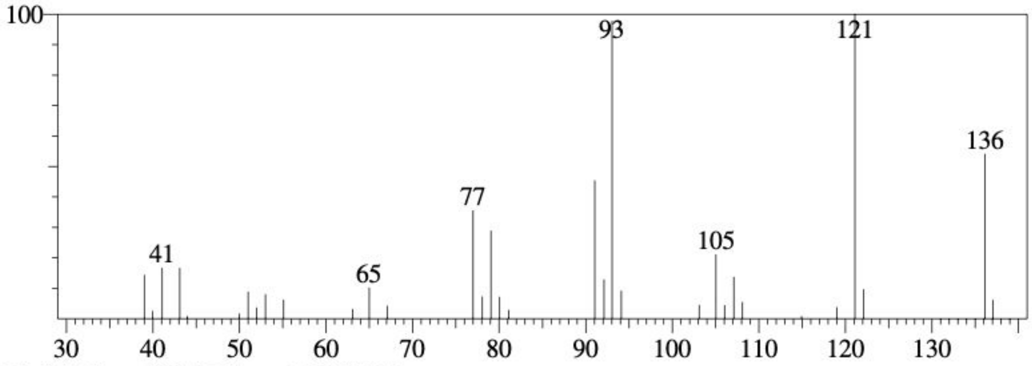

Hit#:1 Entry:9827 Library:NIST11.lib
SI:95 Formula:C10H16 CAS:29050-33-7 MolWeight:136 RetIndex:919
CompName:(+)-4-Carene $$ 4,7,7-Trimethylbicyclo[4.1.0]hept-2-ene # $$

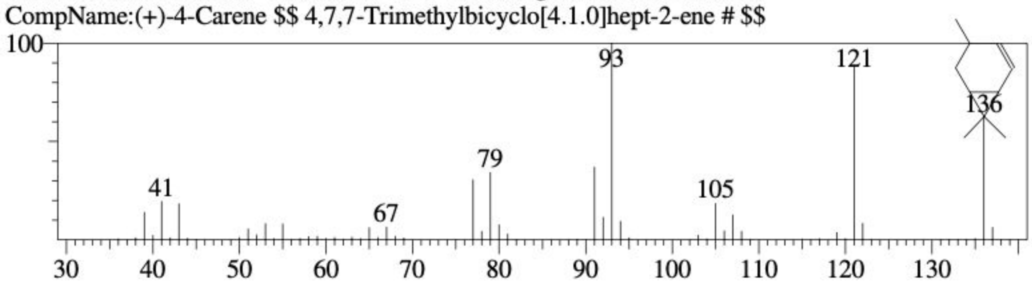

Hit#:2 Entry:277 Library:FFNSC1.3.lib
SI:95 Formula:C10 H16 CAS:99-86-5 MolWeight:136 RetIndex:1018
CompName:Terpinene <alpha->

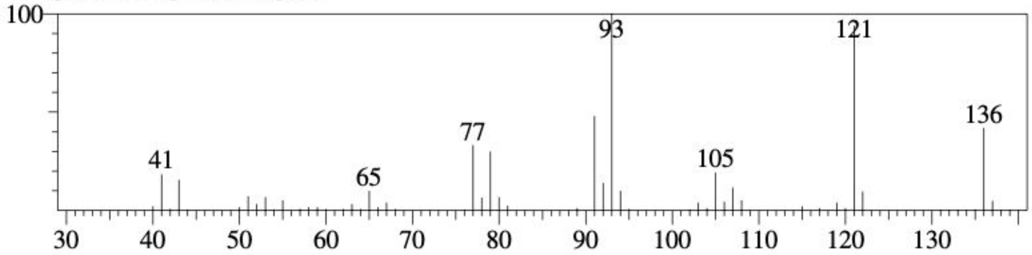

Hit#:3 Entry:25524 Library:WILEY7.LIB
SI:95 Formula:C10 H16 CAS:0-00-0 MolWeight:136 RetIndex:0
CompName:(+)-2-CARENE $$

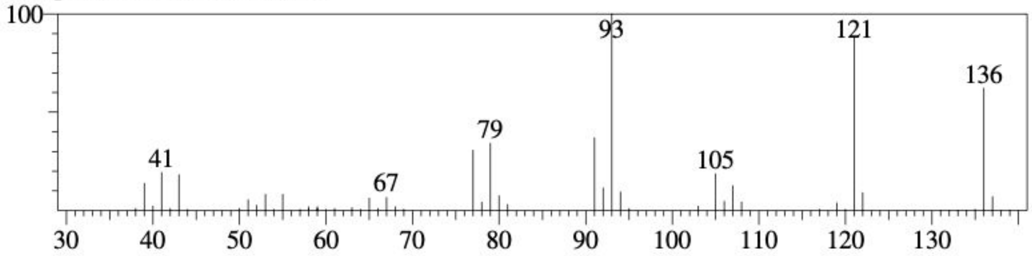

Figure S12. Mass spectrometry figure of the molecular ion associated with Peak 11, corresponding to compound (+)-4-Carene reported by Adams (2007), with a retention time of 8.800min, as well as the product ions (Hit#:1, Hit#:2, and Hit#:3).

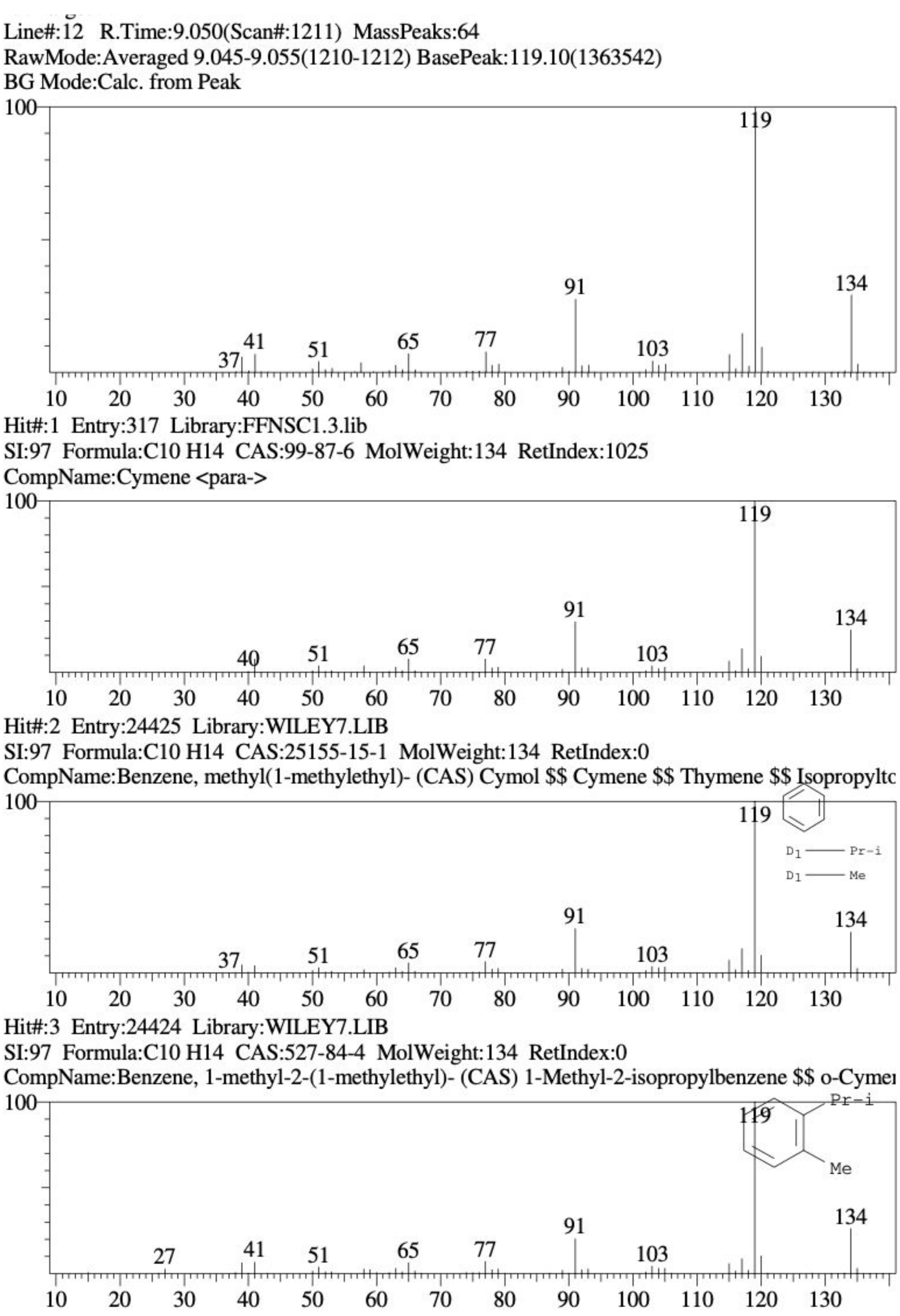

Figure S13. Mass spectrometry figure of the molecular ion associated with Peak 12, corresponding to compound Cymene <para-> reported by Adams (2007), with a retention time of 9.050min, as well as the product ions (Hit#:1, Hit#:2, and Hit#:3).

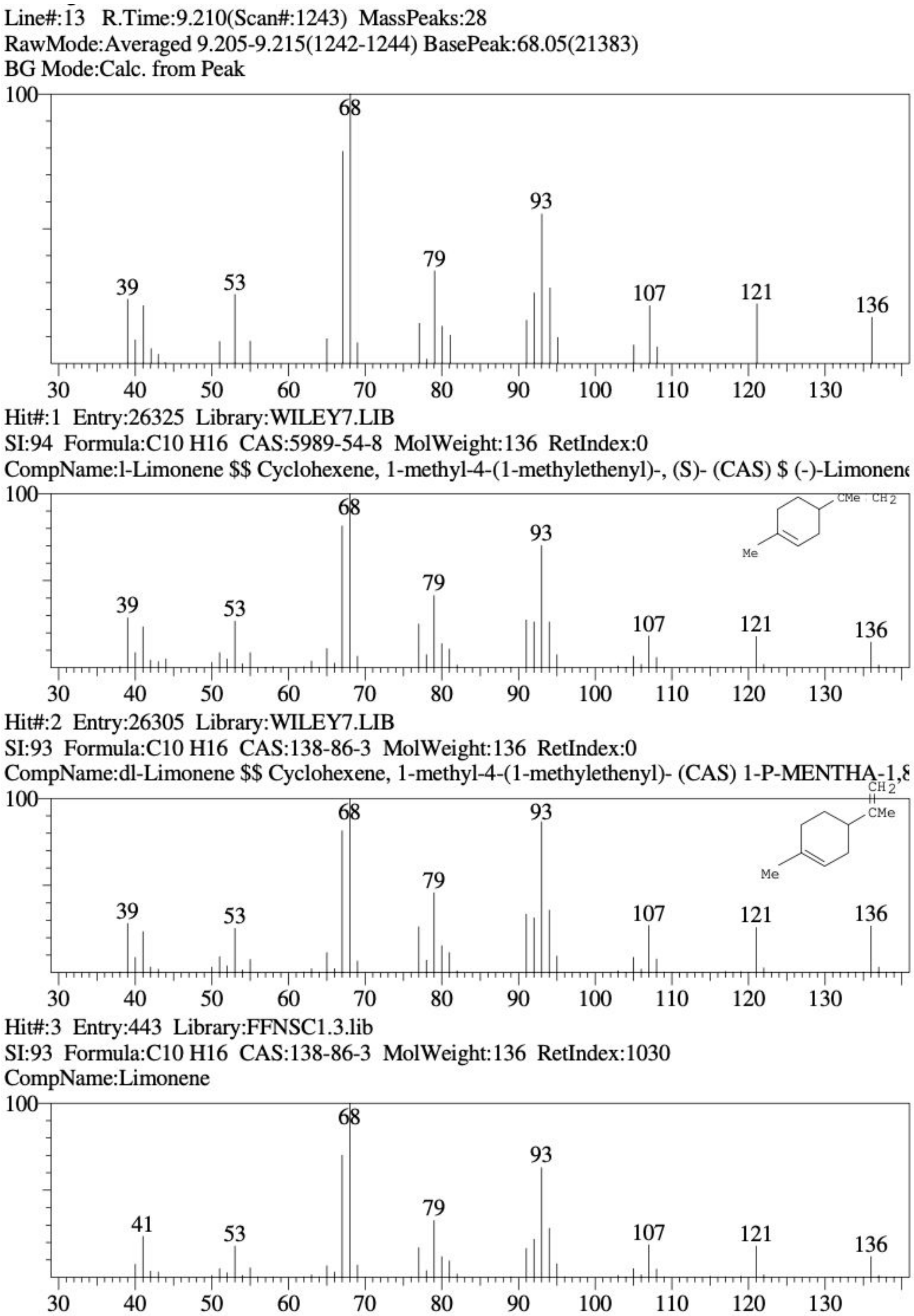

Figure S14. Mass spectrometry figure of the molecular ion associated with Peak 13, corresponding to compound l-Limonene reported by Adams (2007), with a retention time of 9.210min, as well as the product ions (Hit#:1, Hit#:2, and Hit#:3).

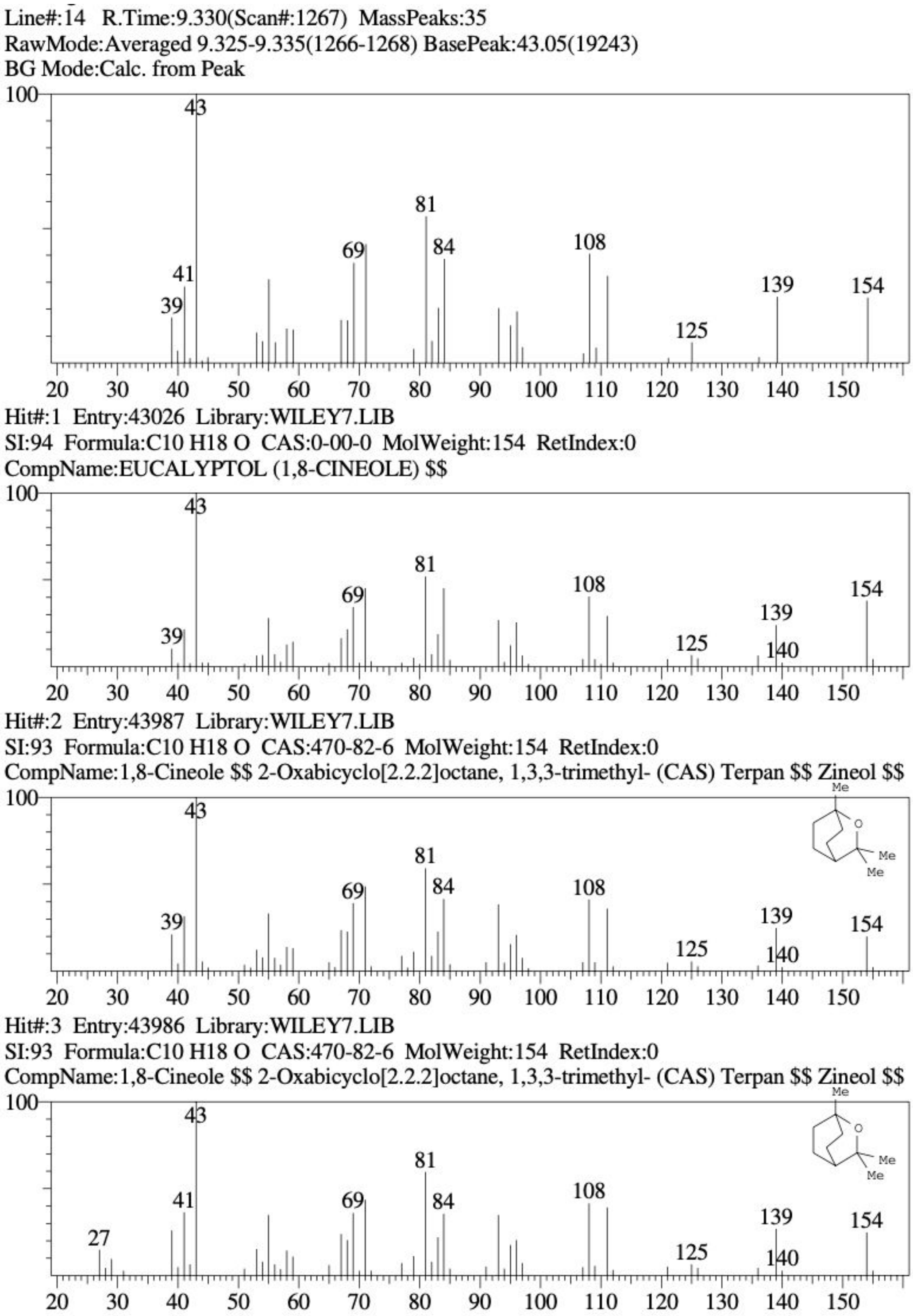

Figure S15. Mass spectrometry figure of the molecular ion associated with Peak 14, corresponding to compound Eucalyptol (1,8-Cineole) reported by Adams (2007), with a retention time of 9.330min, as well as the product ions (Hit#:1, Hit#:2, and Hit#:3).

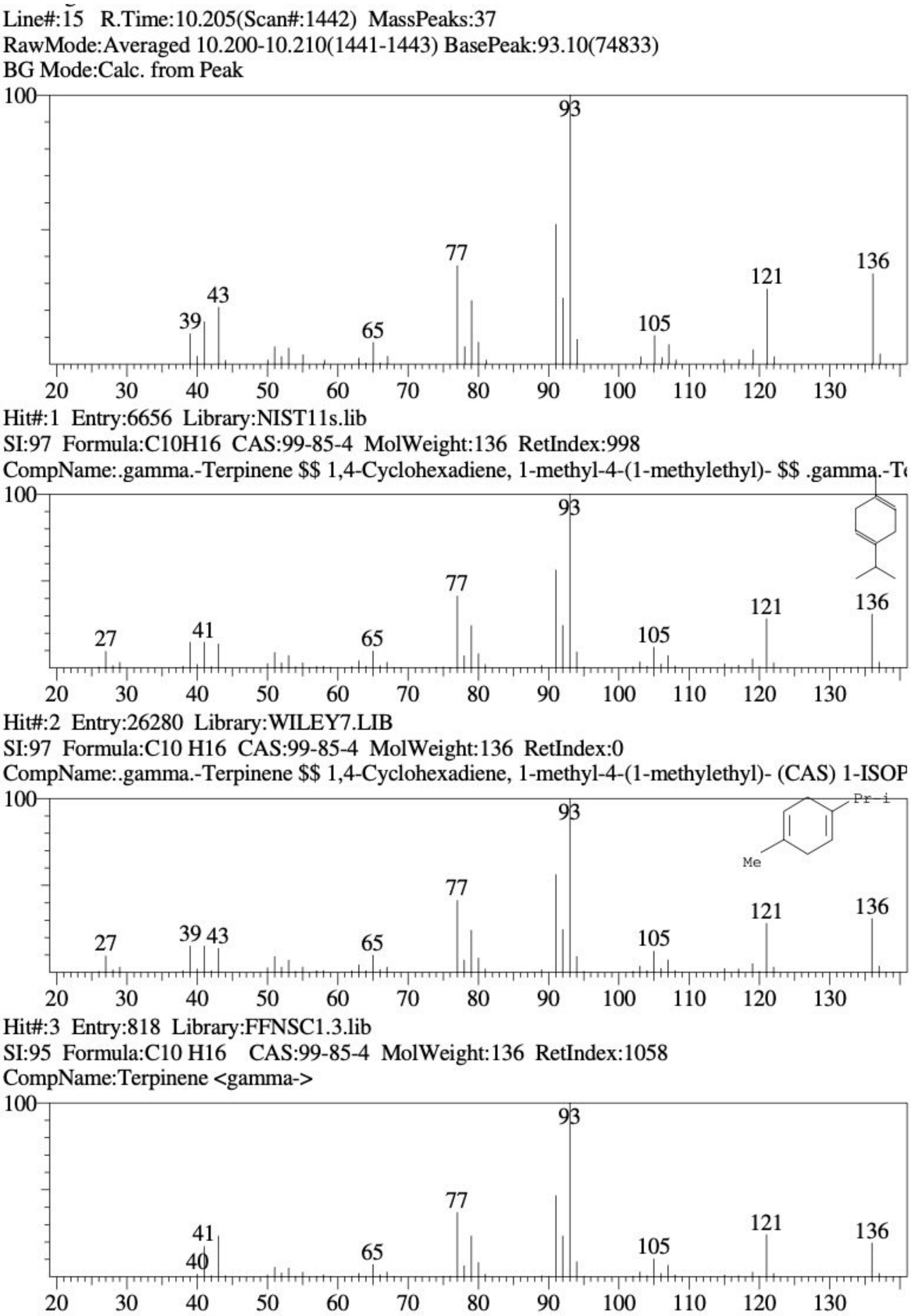

Figure S16. Mass spectrometry figure of the molecular ion associated with Peak 15, corresponding to compound gamma-Terpinene reported by Adams (2007), with a retention time of 10.205min, as well as the product ions (Hit#:1, Hit#:2, and Hit#:3).

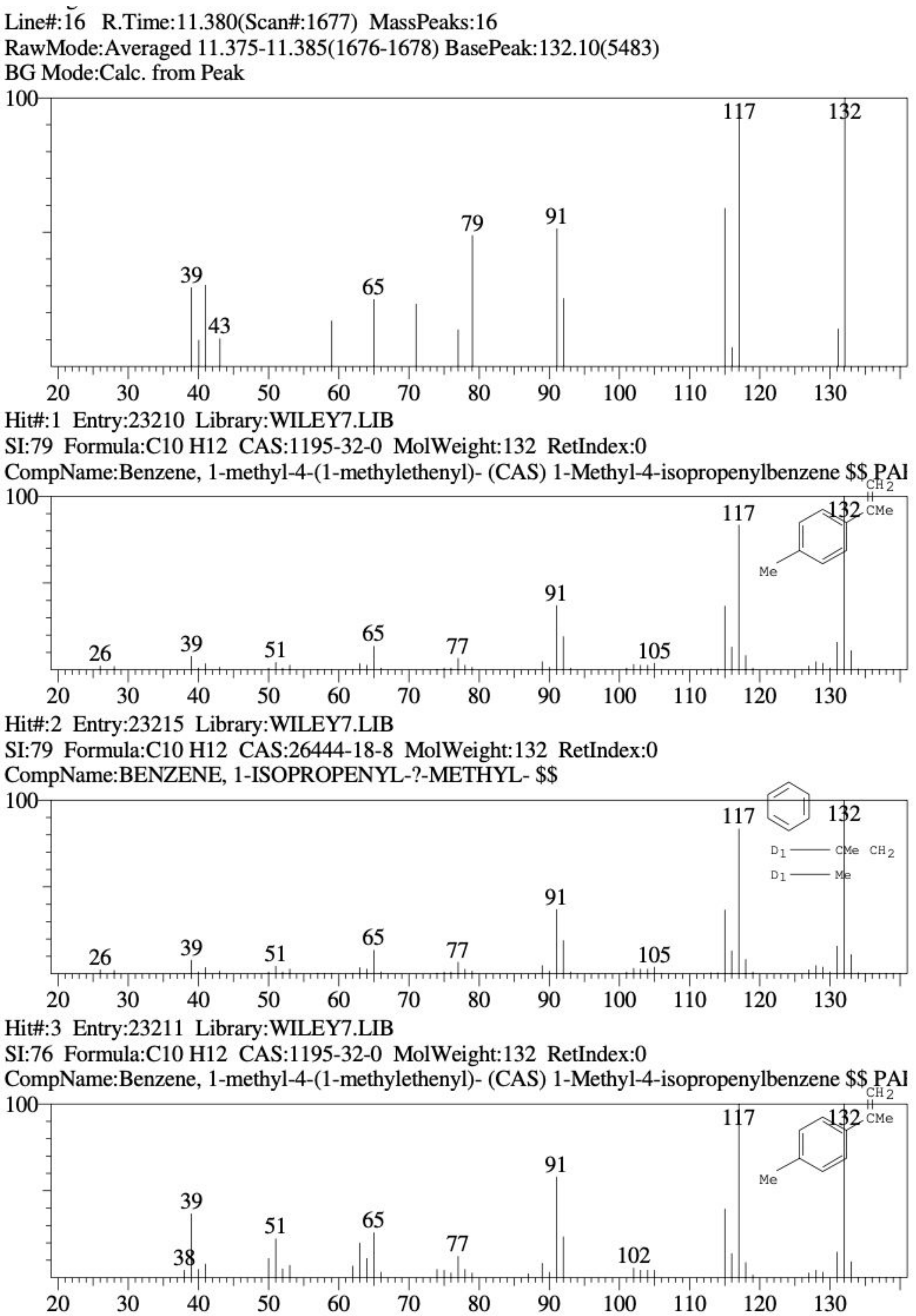

Figure S17. Mass spectrometry figure of the molecular ion associated with Peak 16, corresponding to compound Benzene, 1-methyl-4-(1-methylethenyl)- (CAS) 1-Methyl-4-isopropenylbenzene reported by Adams (2007), with a retention time of 11.380min, as well as the product ions (Hit#:1, Hit#:2, and Hit#:3).

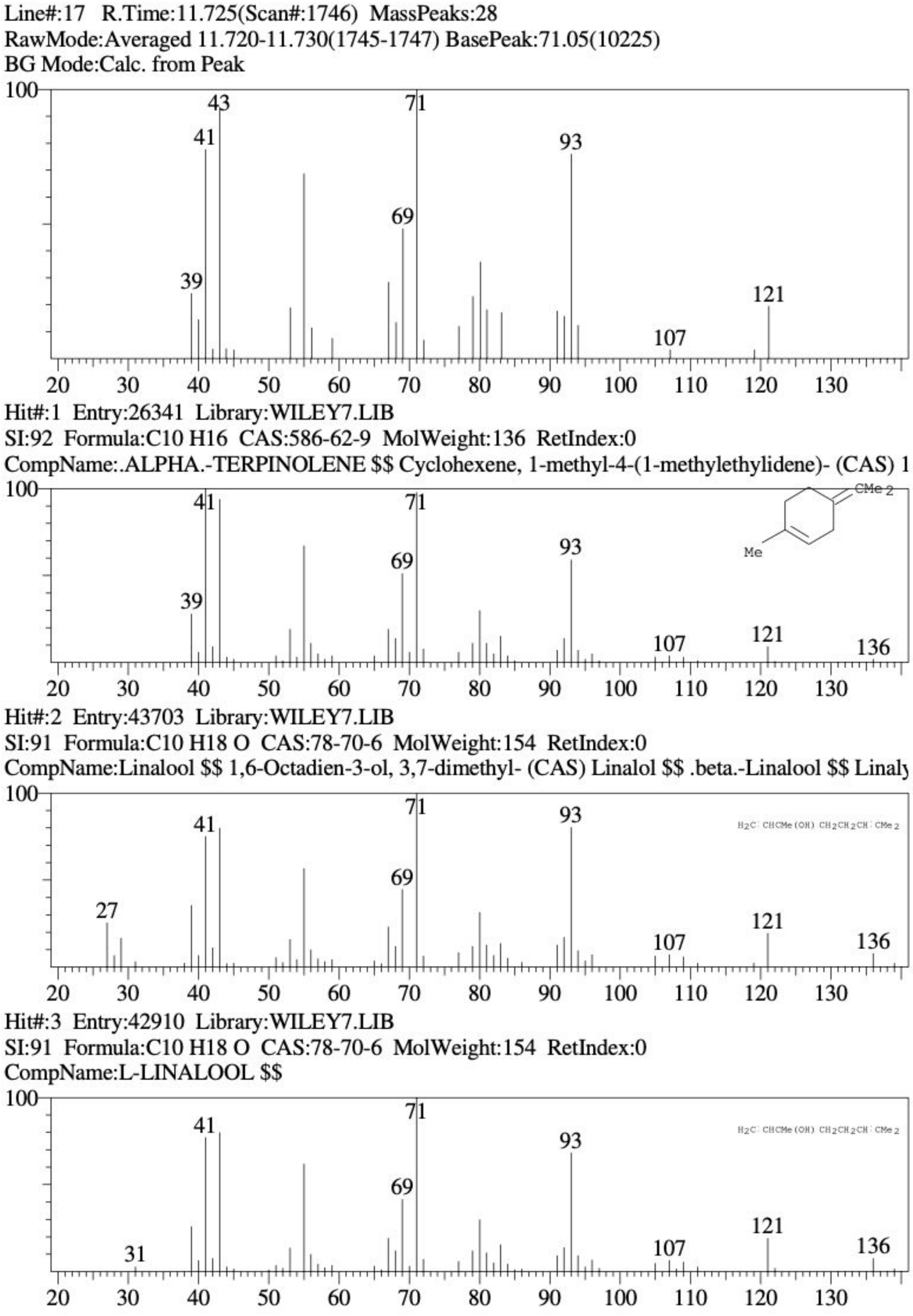

Figure S18. Mass spectrometry figure of the molecular ion associated with Peak 17, corresponding to compound Alpha-Terpinolene reported by Adams (2007), with a retention time of 11.725min, as well as the product ions (Hit#:1, Hit#:2, and Hit#:3).

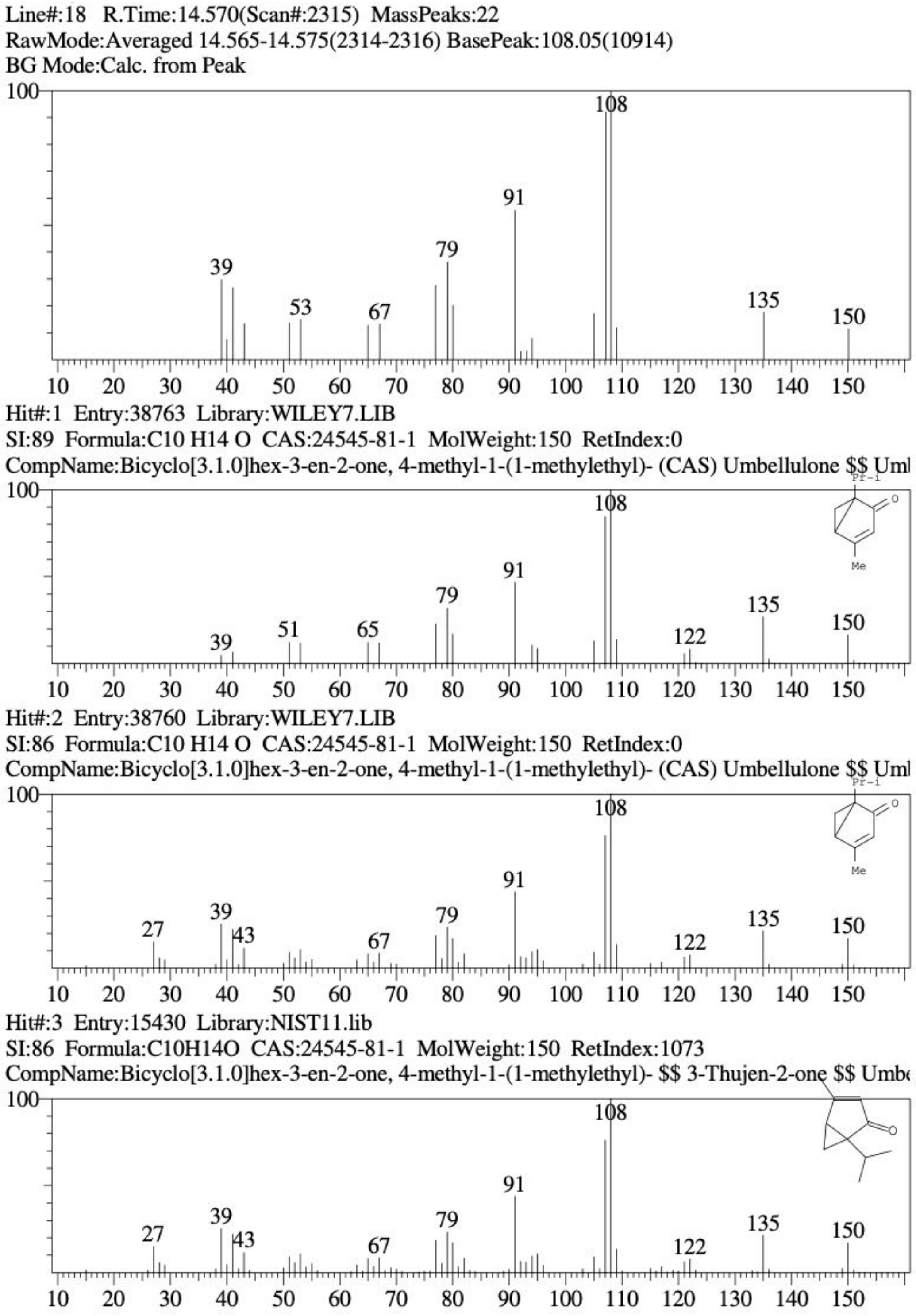

Figure S19. Mass spectrometry figure of the molecular ion associated with Peak 18, corresponding to compound Bicyclo[3.1.0]hex-3-en-2-one, 4-methyl-1-(1-methylethyl)- reported by Adams (2007), with a retention time of 14.570min, as well as the product ions (Hit#:1, Hit#:2, and Hit#:3).

Line#:19 R.Time:15.020(Scan#:2405) MassPeaks:37
RawMode:Averaged 15.015-15.025(2404-2406) BasePeak:71.05(40596)
BG Mode:Calc. from Peak

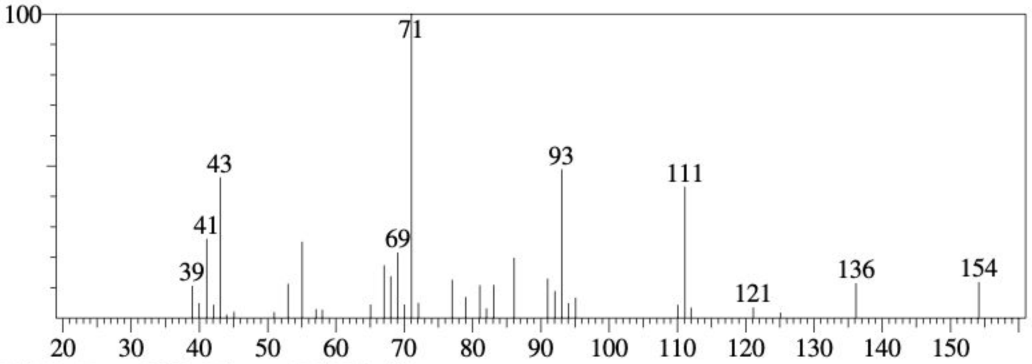

Hit#:1 Entry:9980 Library:NIST11s.lib
SI:95 Formula:C10H18O CAS:562-74-3 MolWeight:154 RetIndex:1137
CompName:Terpinen-4-ol $$ 3-Cyclohexen-1-ol, 4-methyl-1-(1-methylethyl)- $$ p-Menth-1-en-4-o

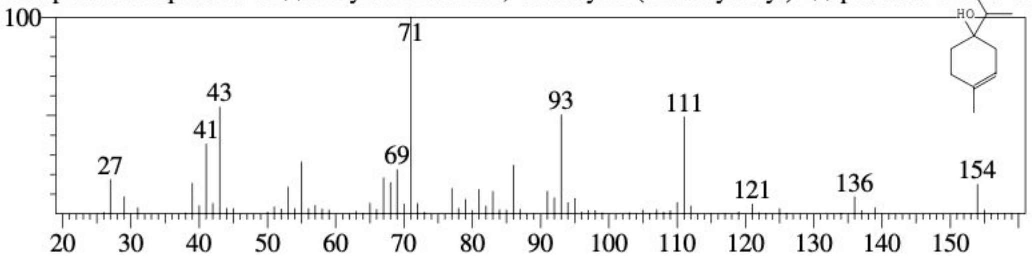

Hit#:2 Entry:43754 Library:WILEY7.LIB
SI:95 Formula:C10 H18 O CAS:562-74-3 MolWeight:154 RetIndex:0
CompName:3-Cyclohexen-1-ol, 4-methyl-1-(1-methylethyl)- (CAS) 4-Terpineol $$ TERPINENE-4-

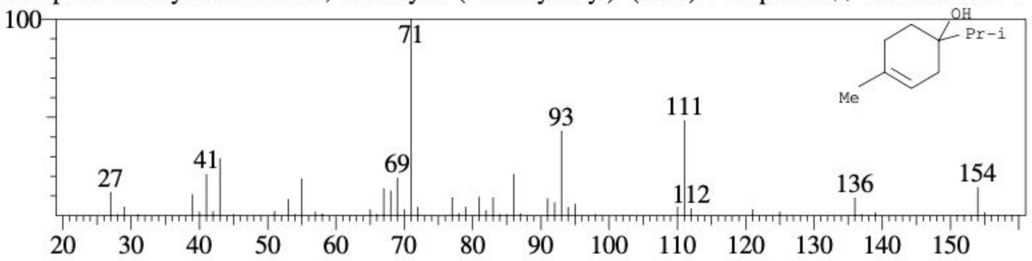

Hit#:3 Entry:43760 Library:WILEY7.LIB
SI:95 Formula:C10 H18 O CAS:562-74-3 MolWeight:154 RetIndex:0
CompName:3-Cyclohexen-1-ol, 4-methyl-1-(1-methylethyl)- (CAS) 4-Terpineol $$ Terpinene-4-ol $

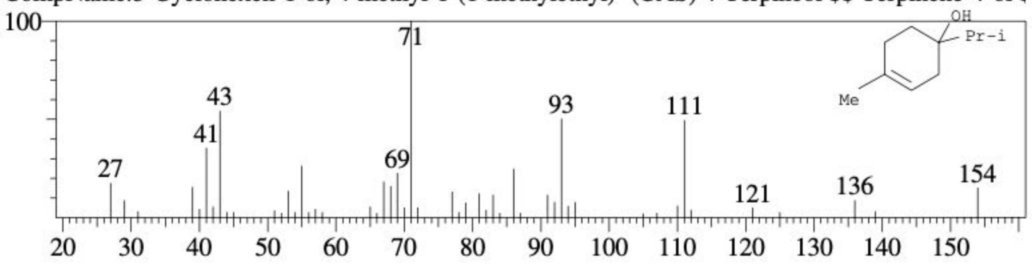

Figure S20. Mass spectrometry figure of the molecular ion associated with Peak 19, corresponding to compound Terpinen-4-ol reported by Adams (2007), with a retention time of 15.020min, as well as the product ions (Hit#:1, Hit#:2, and Hit#:3).

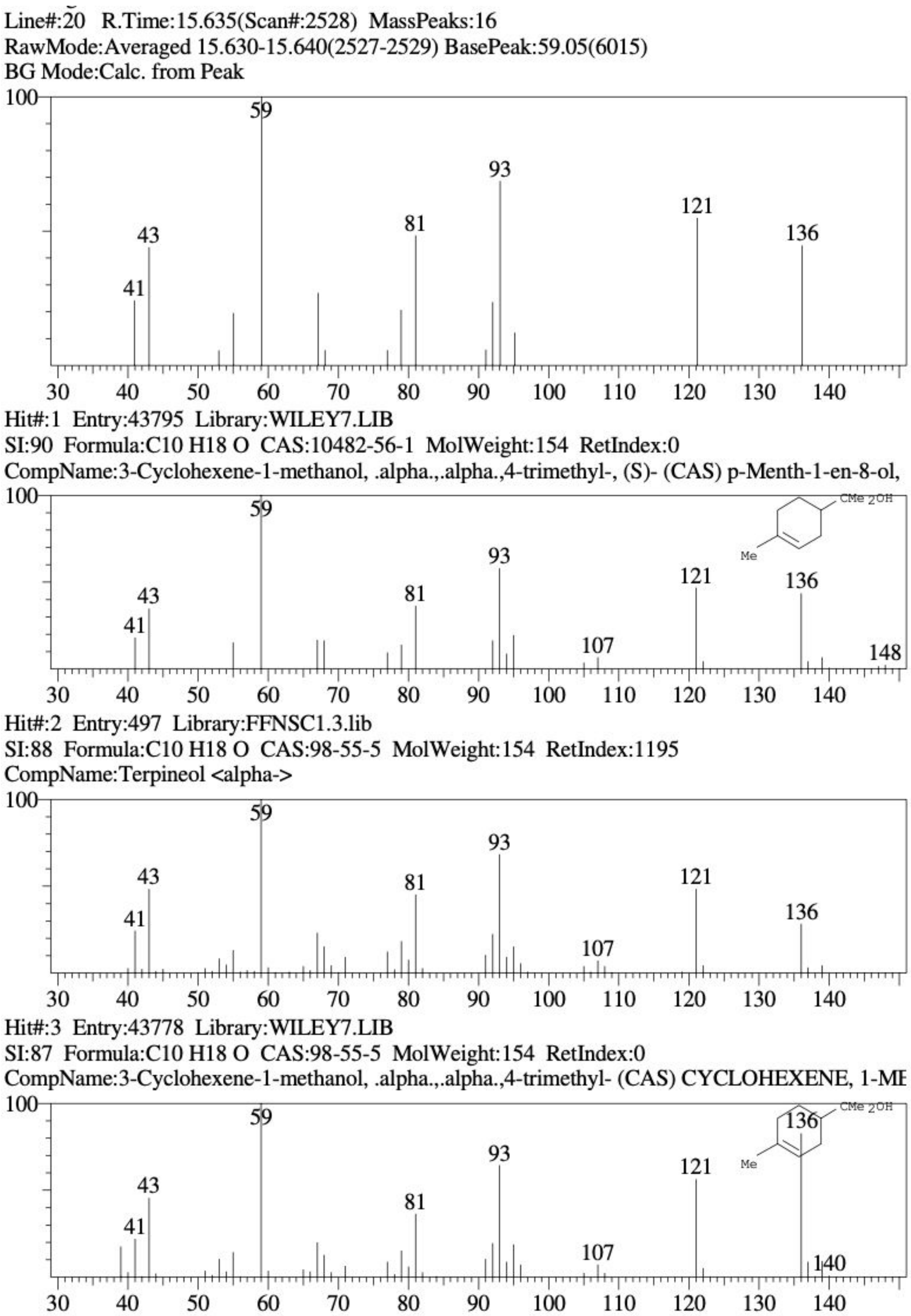

Figure S21. Mass spectrometry figure of the molecular ion associated with Peak 20, corresponding to compound 3-Cyclohexene-1-methanol,alpha,alpha,4-trimethyl, (S)- (CAS) p-Menth-1-en-8-ol, reported by Adams (2007), with a retention time of 15.635min, as well as the product ions (Hit#:1, Hit#:2, and Hit#:3).

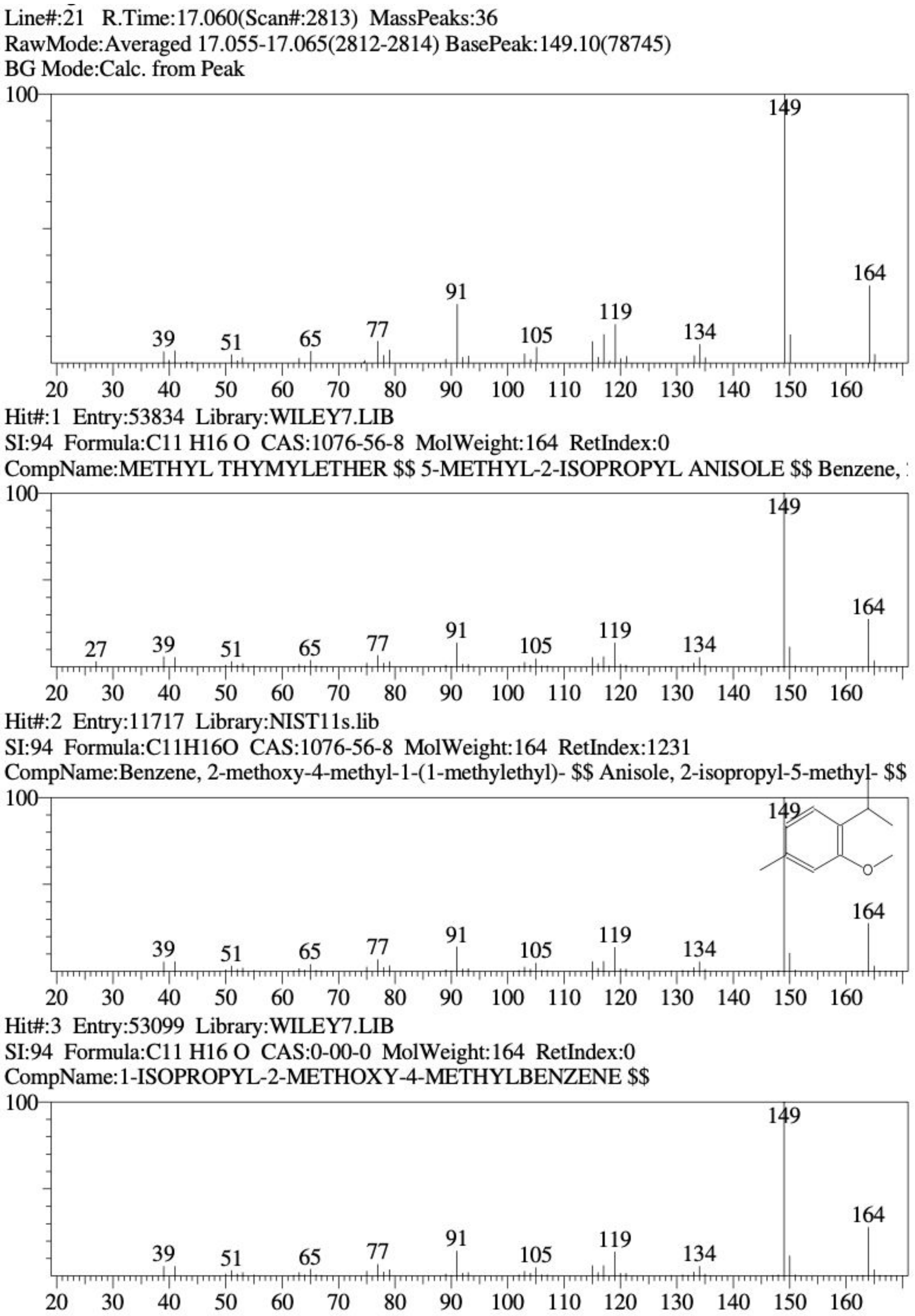

Figure S22. Mass spectrometry figure of the molecular ion associated with Peak 21, corresponding to compound Methyl Thymyl Ether reported by Adams (2007), with a retention time of 17.060min, as well as the product ions (Hit#:1, Hit#:2, and Hit#:3).

Line#:22 R.Time:19.720(Scan#:3345) MassPeaks:99
RawMode:Averaged 19.715-19.725(3344-3346) BasePeak:135.10(5360862)
BG Mode:Calc. from Peak

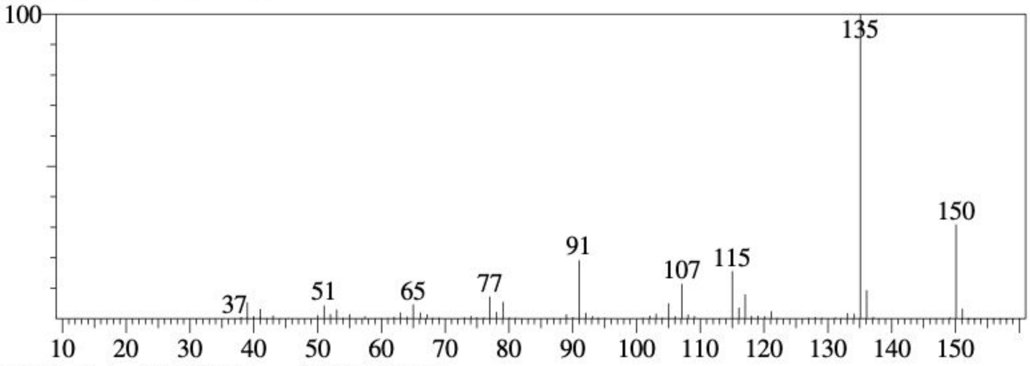

Hit#:1 Entry:38610 Library:WILEY7.LIB
SI:97 Formula:C10 H14 O CAS:89-83-8 MolWeight:150 RetIndex:0
CompName:Phenol, 5-methyl-2-(1-methylethyl)- (CAS) Thymol $$ m-Thymol $$ p-Cymen-3-ol $$

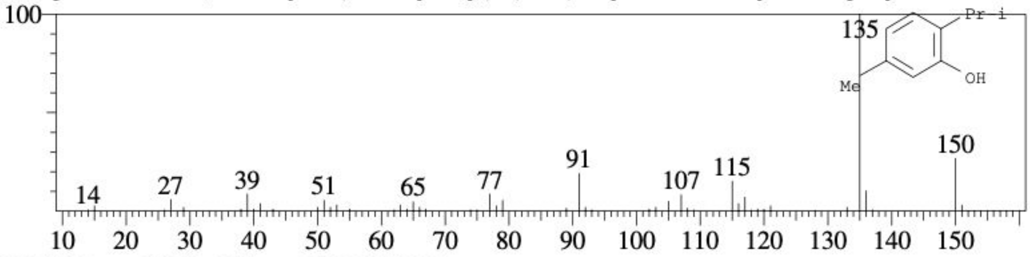

Hit#:2 Entry:38601 Library:WILEY7.LIB
SI:97 Formula:C10 H14 O CAS:89-83-8 MolWeight:150 RetIndex:0
CompName:Phenol, 5-methyl-2-(1-methylethyl)- (CAS) Thymol $$ m-Thymol $$ p-Cymen-3-ol $$

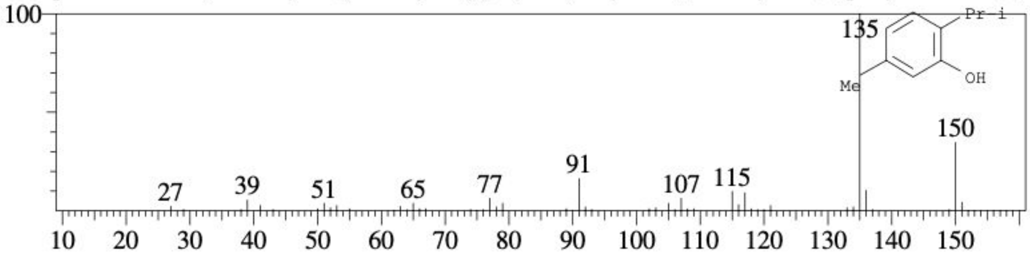

Hit#:3 Entry:9056 Library:NIST11s.lib
SI:96 Formula:C10H14O CAS:89-83-8 MolWeight:150 RetIndex:1262
CompName:Thymol $$ Phenol, 5-methyl-2-(1-methylethyl)- $$ p-Cymen-3-ol $$ Thyme camphor $

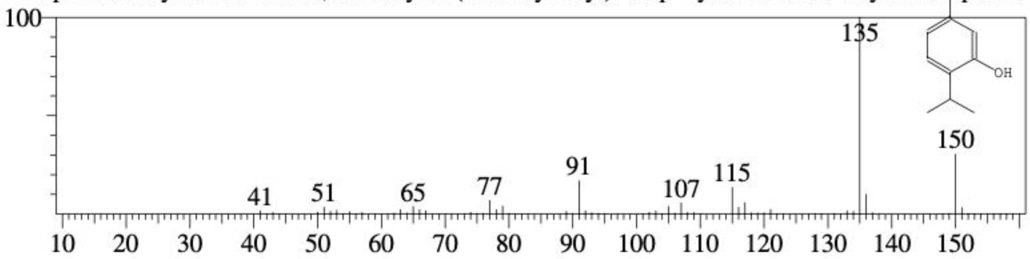

Figure S23. Mass spectrometry figure of the molecular ion associated with Peak 22, corresponding to compound Phenol, 5-methyl-2-(1-methylethyl)- (CAS) Thymol reported by Adams (2007), with a retention time of 19.720min, as well as the product ions (Hit#:1, Hit#:2, and Hit#:3).

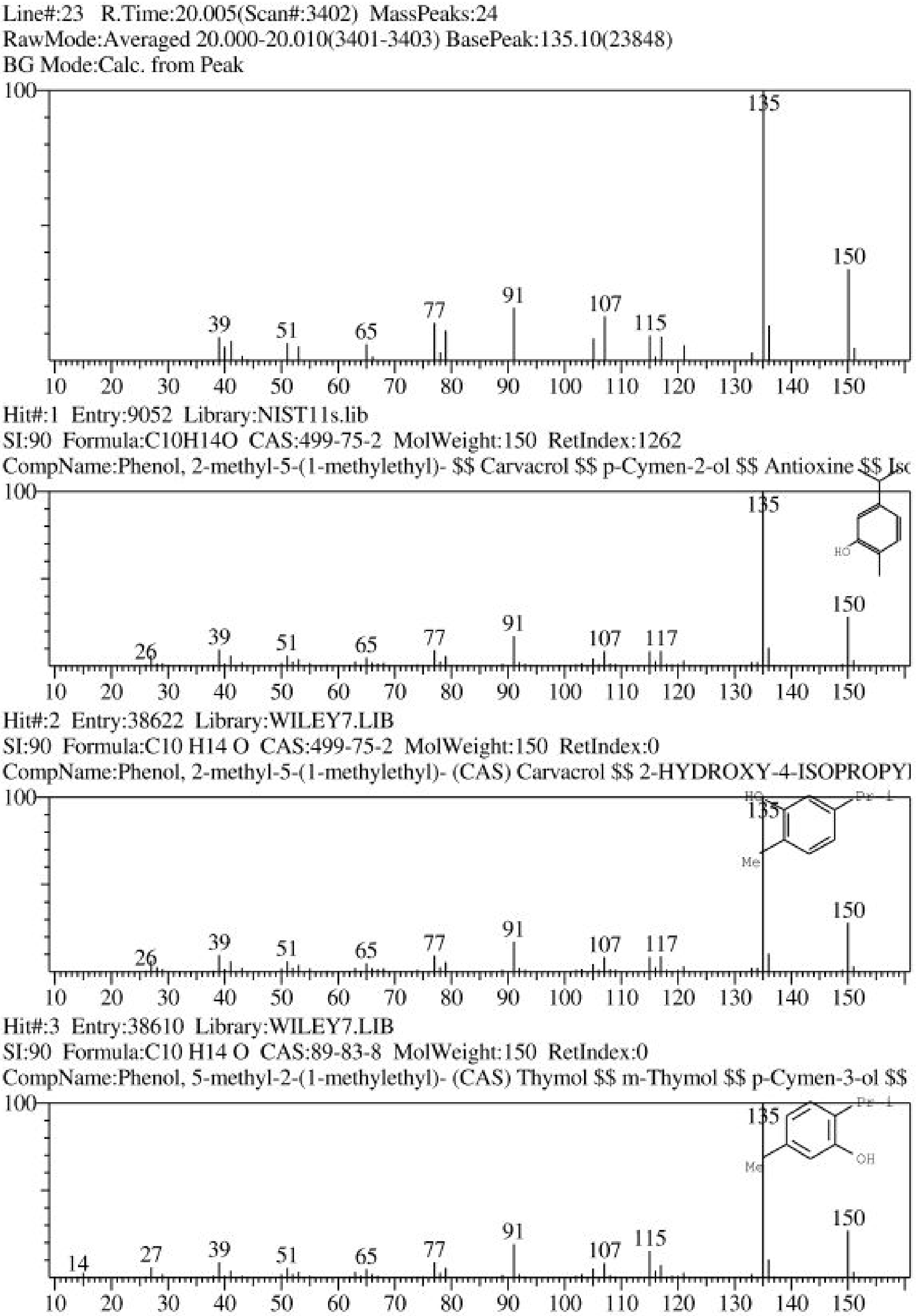

Figure S24. Mass spectrometry figure of the molecular ion associated with Peak 22, corresponding to compound Phenol, 2-methyl-5-(1-methylethyl) - (CAS) Carvacrol reported by Adams (2007), with a retention time of 20.005 min, as well as the product ions (Hit#:1, Hit#:2, and Hit#:3).

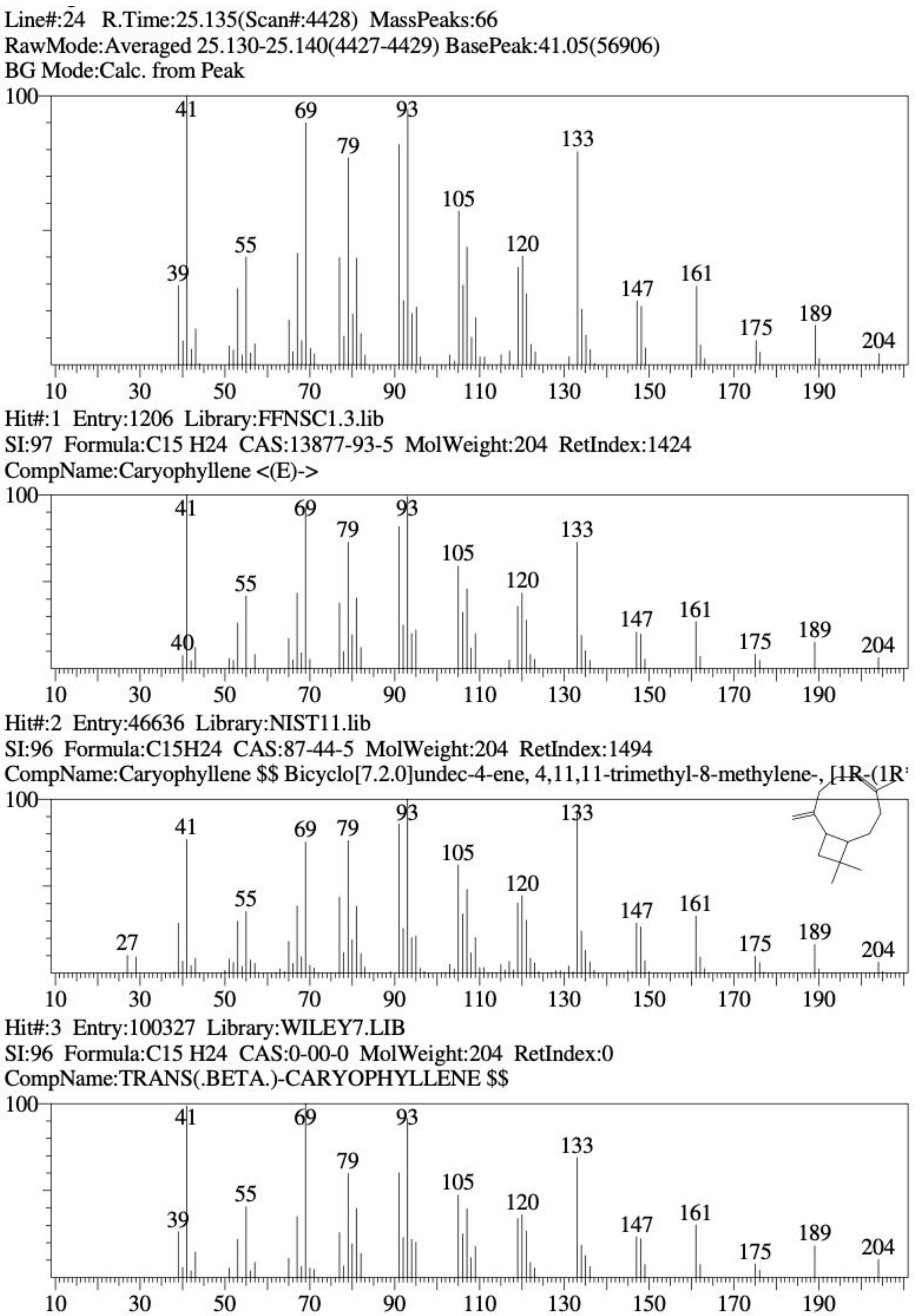

Figure S25. Mass spectrometry figure of the molecular ion associated with Peak 23, corresponding to compound Caryophyllene <(E)->reported by Adams (2007), with a retention time of 25.135min, as well as the product ions (Hit#:1, Hit#:2, and Hit#:3).

Line#:25 R.Time:25.910(Scan#:4583) MassPeaks:36
RawMode:Averaged 25.905-25.915(4582-4584) BasePeak:41.05(5259)
BG Mode:Calc. from Peak

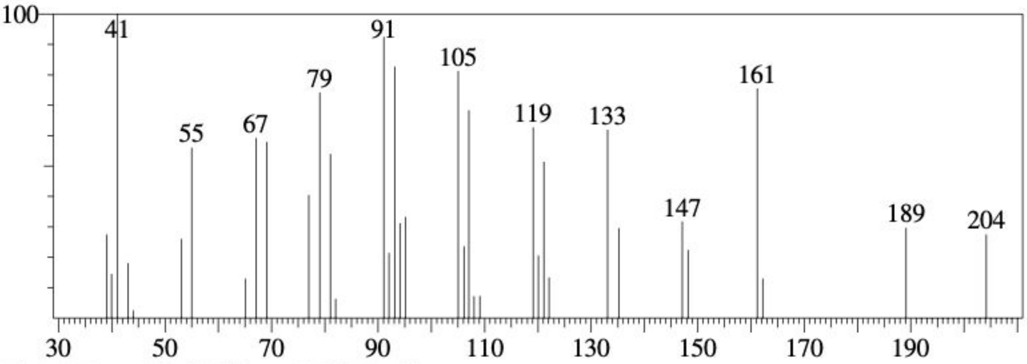

Hit#:1 Entry:18061 Library:NIST11s.lib
SI:92 Formula:C15H24 CAS:25246-27-9 MolWeight:204 RetIndex:1386
CompName:Alloaromadendrene $$ 1H-Cycloprop[e]azulene, decahydro-1,1,7-trimethyl-4-methylen

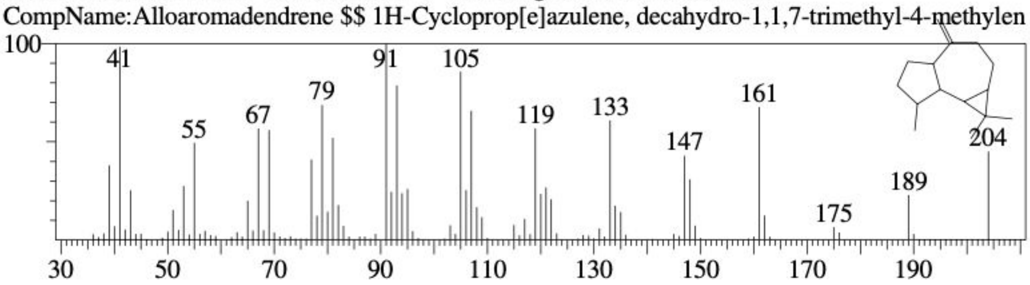

Hit#:2 Entry:101016 Library:WILEY7.LIB
SI:92 Formula:C15 H24 CAS:25246-27-9 MolWeight:204 RetIndex:0
CompName:Alloaromadendrene $$ 1H-Cycloprop[e]azulene, decahydro-1,1,7-trimethyl-4-methylen

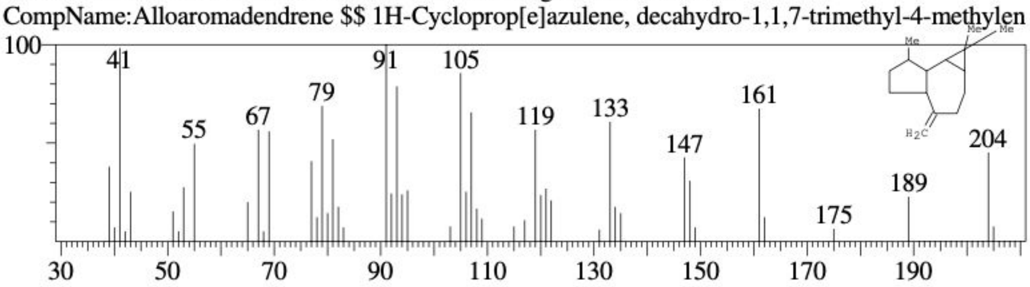

Hit#:3 Entry:101007 Library:WILEY7.LIB
SI:92 Formula:C15 H24 CAS:489-39-4 MolWeight:204 RetIndex:0
CompName:(+)-Aromadendrene $$ 1H-Cycloprop[e]azulene, decahydro-1,1,7-trimethyl-4-methylen

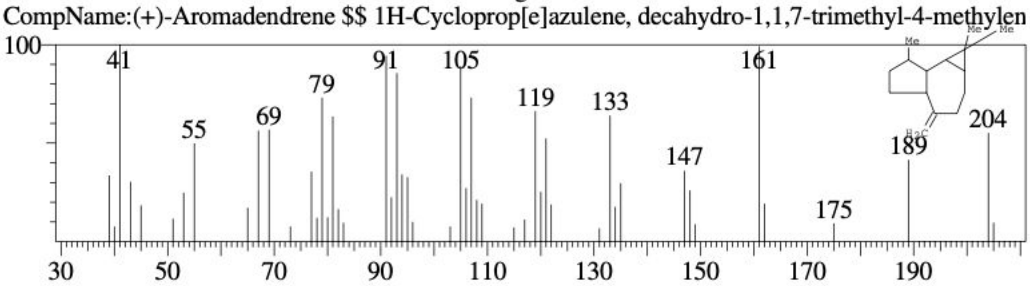

Figure S26. Mass spectrometry figure of the molecular ion associated with Peak 25, corresponding to compound Alloaromadendrene reported by Adams (2007), with a retention time of 25.910min, as well as the product ions (Hit#:1, Hit#:2, and Hit#:3).

Line#:26 R.Time:26.610(Scan#:4723) MassPeaks:21
RawMode:Averaged 26.605-26.615(4722-4724) BasePeak:93.10(8150)
BG Mode:Calc. from Peak

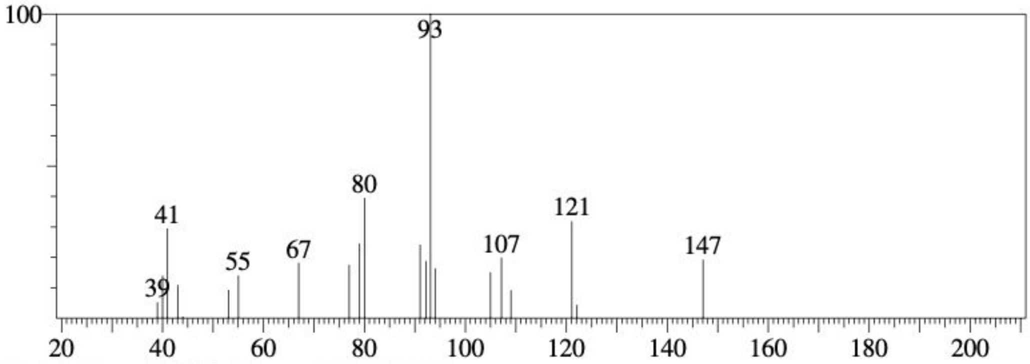

Hit#:1 Entry:100910 Library:WILEY7.LIB
SI:85 Formula:C15 H24 CAS:17066-67-0 MolWeight:204 RetIndex:0
CompName:.beta.-Selinene $$ Naphthalene, decahydro-4a-methyl-1-methylene-7-(1-methylethenyl)

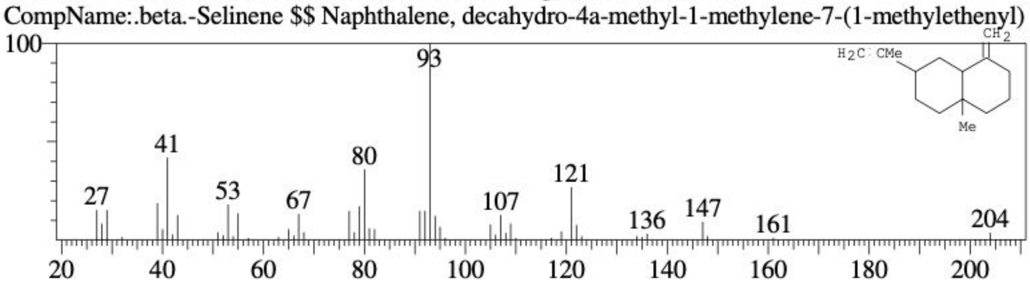

Hit#:2 Entry:100913 Library:WILEY7.LIB
SI:85 Formula:C15 H24 CAS:17066-67-0 MolWeight:204 RetIndex:0
CompName:.beta.-Selinene $$ Naphthalene, decahydro-4a-methyl-1-methylene-7-(1-methylethenyl)

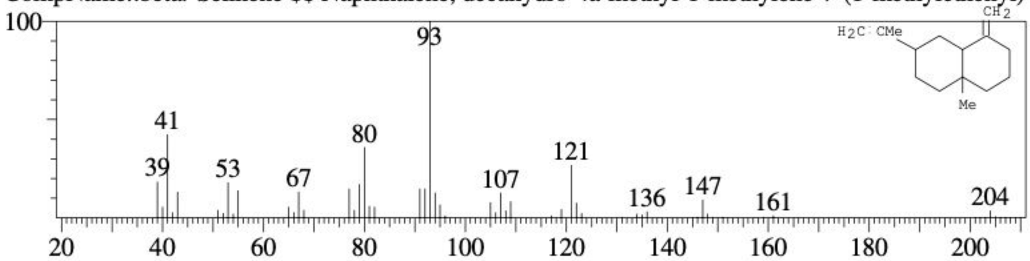

Hit#:3 Entry:100734 Library:WILEY7.LIB
SI:85 Formula:C15 H24 CAS:6753-98-6 MolWeight:204 RetIndex:0
CompName:.alpha.-Humulene $$ 1,4,8-Cycloundecatriene, 2,6,6,9-tetramethyl-, (E,E,E)- (CAS) 4,7

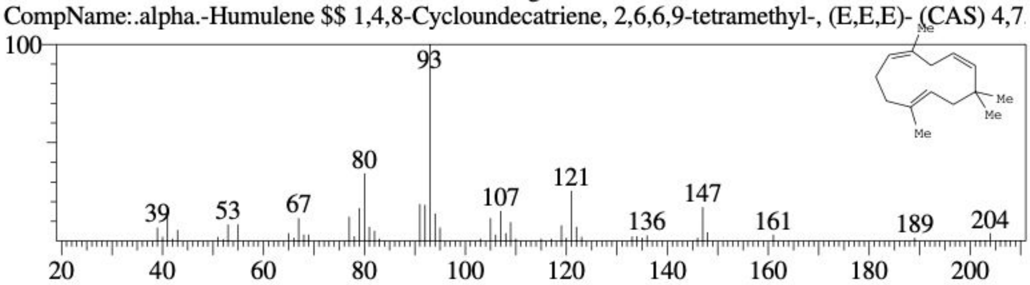

Figure S27. Mass spectrometry figure of the molecular ion associated with Peak 26, corresponding to compound beta-Selinene reported by Adams (2007), with a retention time of 26.610min, as well as the product ions (Hit#:1, Hit#:2, and Hit#:3).

Line#:27 R.Time:28.060(Scan#:5013) MassPeaks:21
RawMode:Averaged 28.055-28.065(5012-5014) BasePeak:107.10(3662)
BG Mode:Calc. from Peak

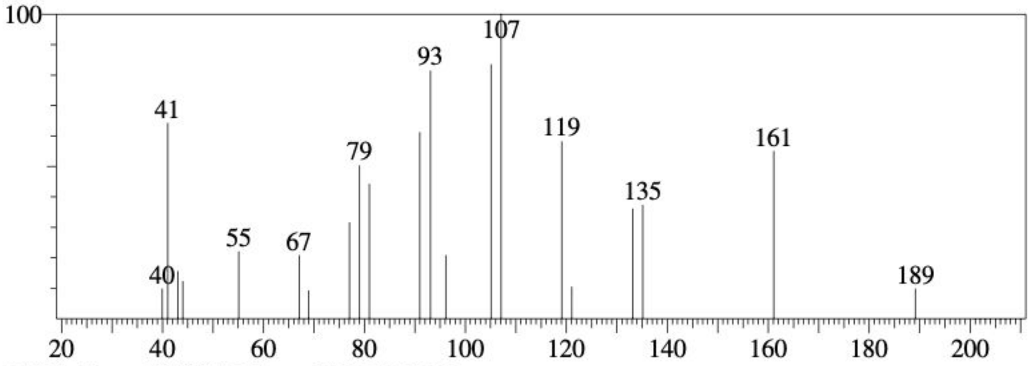

Hit#:1 Entry:101024 Library:WILEY7.LIB
SI:87 Formula:C15 H24 CAS:21747-46-6 MolWeight:204 RetIndex:0
CompName:Ledene $$ 1H-Cycloprop[e]azulene, 1a,2,3,5,6,7,7a,7b-octahydro-1,1,4,7-tetramethyl-,

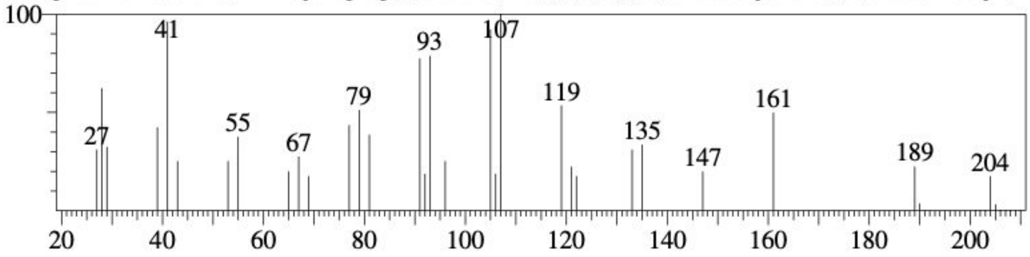

Hit#:2 Entry:100940 Library:WILEY7.LIB
SI:79 Formula:C15 H24 CAS:4630-07-3 MolWeight:204 RetIndex:0
CompName:valencene 2 $$

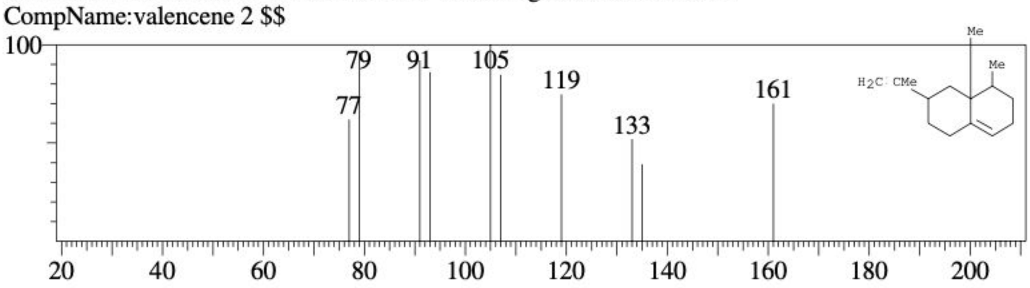

Hit#:3 Entry:1038 Library:FFNSC1.3.lib
SI:79 Formula:C15 H24 CAS:74409-93-1 MolWeight:204 RetIndex:1491
CompName:Viridiflorene

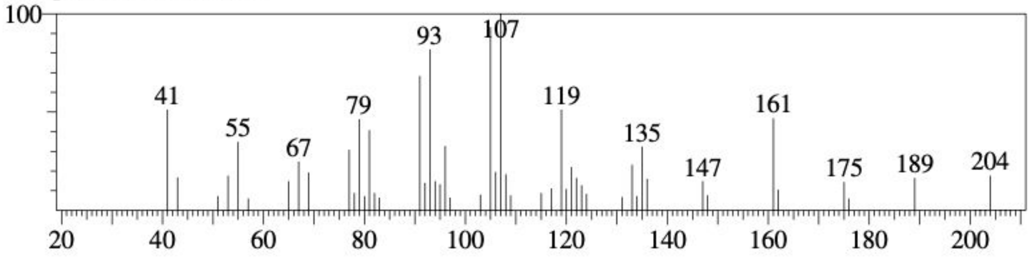

Figure S28. Mass spectrometry figure of the molecular ion associated with Peak 27, corresponding to compound Ledene reported by Adams (2007), with a retention time of 28.060min, as well as the product ions (Hit#:1, Hit#:2, and Hit#:3).

Line#:28 R.Time:31.650(Scan#:5731) MassPeaks:49
RawMode:Averaged 31.645-31.655(5730-5732) BasePeak:41.05(11678)
BG Mode:Calc. from Peak

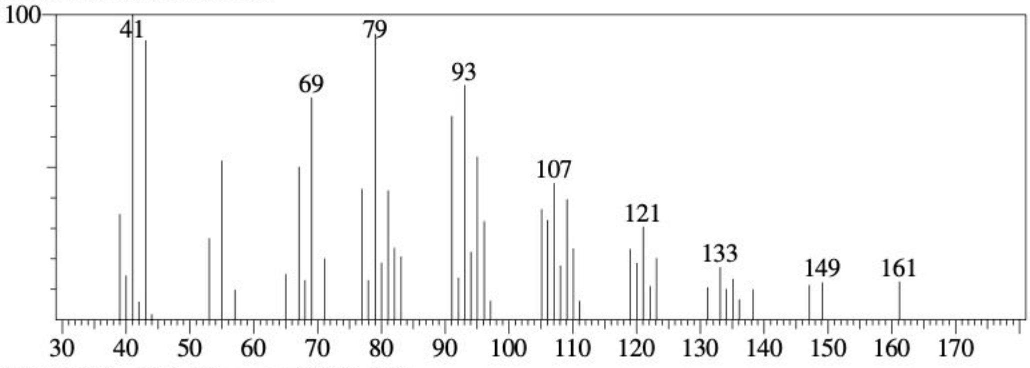

Hit#:1 Entry:145 Library:FFNSC1.3.lib
SI:95 Formula:C15 H24 O CAS:17627-43-9 MolWeight:220 RetIndex:1587
CompName:Caryophyllene oxide

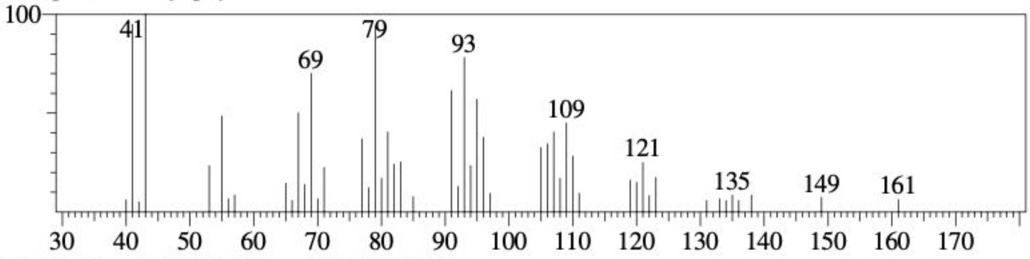

Hit#:2 Entry:121056 Library:WILEY7.LIB
SI:95 Formula:C15 H24 O CAS:1139-30-6 MolWeight:220 RetIndex:0
CompName:(-)-Caryophyllene oxide $$ (-)-5-Oxatricyclo[8.2.0.0(4,6)]dodecane,,12-trimethyl-9-meth

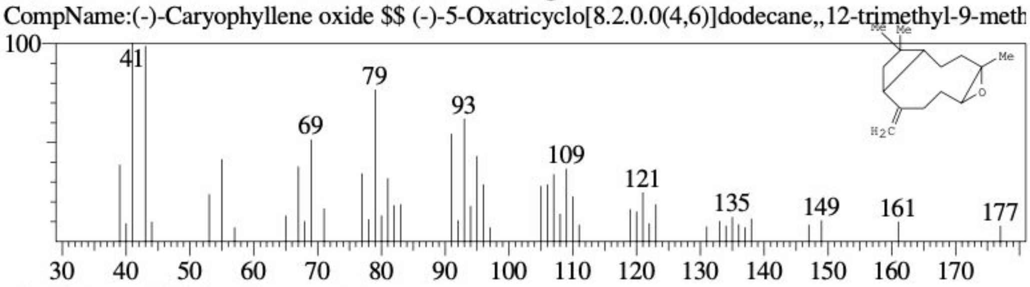

Hit#:3 Entry:121057 Library:WILEY7.LIB
SI:94 Formula:C15 H24 O CAS:1139-30-6 MolWeight:220 RetIndex:0
CompName:(-)-Caryophyllene oxide $$ (-)-5-Oxatricyclo[8.2.0.0(4,6)]dodecane,,12-trimethyl-9-meth

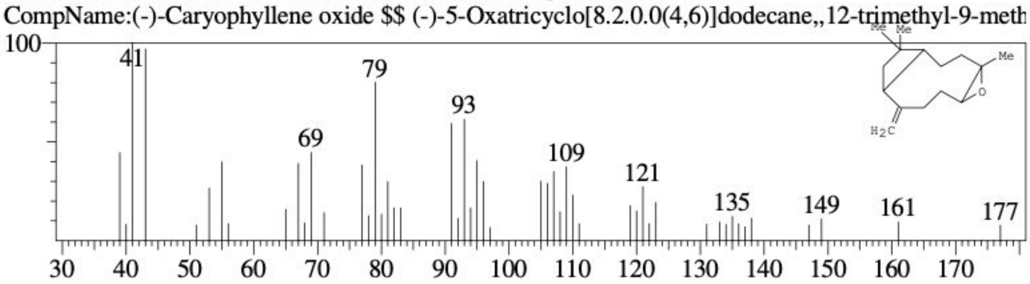

Figure S29. Mass spectrometry figure of the molecular ion associated with Peak 28, corresponding to compound Caryophyllene oxide reported by Adams (2007), with a retention time of 31.650 as well as the product ions (Hit#:1, Hit#:2, and Hit#:3).

Table S1. Chemical composition of *Lippia origanoides* Kunth Essential Oil obtained from GC-MS Analysis (Shimadzu GCMS QP-2010, Japan) using Homologous Alkanes (C_8–C_{20}) for Constituent Identification as reported by Adams (2007). The number of peaks, retention time (R. Time), peak area, compound name, and mass-to-charge ratio molecular ion (Base m/z) are available.

Peak Report TIC

Peak#	R.Time	Area	Area%	Name	Base m/z
1	3.076	545353	0.59	Propanoic acid, 2-methyl-, methyl ester (CAS) Methyl iso	43.1
2	3.131	235430	0.26	Butane, 1-ethoxy-	59.1
3	3.365	159581	0.17	Hexane, 2,5-dimethyl- (CAS) 2,5-Dimethylhexane	57.1
4	3.625	33455	0.04	2-Hexene, 2,5-dimethyl-	69.0
5	6.249	67025	0.07	.alpha.-Thujene	93.1
6	6.456	368760	0.40	.ALPHA.-PINENE, (-)-	93.1
7	7.588	60743	0.07	3-Heptanone	57.1
8	7.640	44113	0.05	Sabinene	93.1
9	7.888	1953168	2.12	.beta.-Myrcene	41.0
10	8.549	92395	0.10	.DELTA.3-Carene	93.1
11	8.801	564259	0.61	(+)-4-Carene	121.1
12	9.048	9818262	10.67	Cymene <para->	119.1
13	9.210	351792	0.38	l-Limonene	68.0
14	9.330	437986	0.48	EUCALYPTOL (1,8-CINEOLE)	43.1
15	10.204	928922	1.01	.gamma.-Terpinene	93.1
16	11.380	82790	0.09	Benzene, 1-methyl-4-(1-methylethenyl)- (CAS) 1-Methyl-	132.1
17	11.727	217150	0.24	.ALPHA.-TERPINOLENE	71.0
18	14.571	223658	0.24	Bicyclo[3.1.0]hex-3-en-2-one, 4-methyl-1-(1-methylethyl)	108.1
19	15.021	714897	0.78	Terpinen-4-ol	71.0
20	15.635	90552	0.10	3-Cyclohexene-1-methanol, .alpha.,.alpha.,4-trimethyl-, (S	59.1
21	17.058	695983	0.76	METHYL THYMYLETHER	149.1
22	19.718	70019898	76.06	Phenol, 5-methyl-2-(1-methylethyl)- (CAS) Thymol	135.1
23	20.005	233924	0.25	Phenol, 2-methyl-5-(1-methylethyl)-	135.1
24	25.137	3012198	3.27	Caryophyllene <(E)->	41.1
25	25.909	249414	0.27	Alloaromadendrene	41.1
26	26.608	101210	0.11	.beta.-Selinene	93.1
27	28.062	95786	0.10	Ledene	107.1
28	31.648	659650	0.72	Caryophyllene oxide	41.1
		92058354	100.00		

Table S2. Calculation of Kováts and Linear Retention Indices (IRL) from Primary GC-MS Data (Shimadzu GCMS QP-2010, Japan) of *Lippia origanoides* Kunth Essential Oil Using Homologous Alkanes (C_8–C_{20}) Based on Adams (2007). Peaks 1–4 correspond to the detection of the solvents used in the method and do not have corresponding values in the alkane series, preventing IRL determination.

Peak	t_r	C_n	t_r C_n	t_r C_{n+1}	IRL
1	3.076	-	-	-	-
2	3.131	-	-	-	-
3	3.365	-	-	-	-
4	3.625	-	-	-	-
5	6.243	9	5.635	8.238	923
6	6.456	9	5.635	8.238	932
7	7.588	9	5.635	8.238	975
8	7.640	9	5.635	8.238	977
9	7.888	9	5.635	8.238	987
10	8.549	10	8.238	11.777	1009
11	8.801	10	8.238	11.777	1016
12	9.048	10	8.238	11.777	1023
13	9.210	10	8.238	11.777	1027
14	9.330	10	8.238	11.777	1031
15	10.204	10	8.238	11.777	1056
16	11.380	10	8.238	11.777	1089
17	11.727	10	8.238	11.777	1099
18	14.571	11	11.777	15.892	1168
19	15.021	11	11.777	15.892	1179
20	15.635	11	11.777	15.892	1194
21	17.058	12	15.892	20.196	1227
22	19.718	12	15.892	20.196	1289
23	20.005	12	15.892	20.196	1296
24	25.137	14	24.468	28.596	1416
25	25.909	14	24.468	28.596	1435
26	26.608	14	24.468	28.596	1452
27	28.062	14	24.468	28.596	1487
28	31.648	15	28.596	32.548	1577

t_r: retention time of the essential oil compound; **C_n:** number of carbon atoms in the preceding alkane; **t_r C_n:** retention time of alkane N with t_r preceding a specific essential oil compound; **t_r C_{n+1}:** retention time of alkane N with t_r following a specific essential oil compound; **IRL:** linear retention index; **(-)**: nonexistent or undetermined value.
